# Social Vulnerability, Intervention Utilization, and Outcomes in US Adults Hospitalized With Influenza

**DOI:** 10.1001/jamanetworkopen.2024.48003

**Published:** 2024-11-04

**Authors:** Katherine Adams, Kimberly Yousey-Hindes, Catherine H. Bozio, Seema Jain, Pam Daily Kirley, Isaac Armistead, Nisha B. Alden, Kyle P. Openo, Lucy S. Witt, Maya L. Monroe, Sue Kim, Anna Falkowski, Ruth Lynfield, Melissa McMahon, Marisa R. Hoffman, Yomei P. Shaw, Nancy L. Spina, Adam Rowe, Christina B. Felsen, Erin Licherdell, Krista Lung, Eli Shiltz, Ann Thomas, H. Keipp Talbot, William Schaffner, Melanie T. Crossland, Kristen P. Olsen, Larry W. Chang, Charisse N. Cummings, Mark W. Tenforde, Shikha Garg, James L. Hadler, Alissa O’Halloran

**Affiliations:** Influenza Division, Centers for Disease Control and Prevention, Atlanta, Georgia; Connecticut Emerging Infections Program, Yale School of Public Health, New Haven; Influenza Division, Centers for Disease Control and Prevention, Atlanta, Georgia; California Department of Public Health, Richmond; California Emerging Infections Program, Oakland; Colorado Department of Public Health and Environment, Denver; Colorado Department of Public Health and Environment, Denver; Division of Infectious Diseases, School of Medicine, Emory University, Atlanta, Georgia; Georgia Emerging Infections Program, Georgia Department of Public Health, Atlanta; Atlanta Veterans Affairs Medical Center, Decatur, Georgia; Georgia Emerging Infections Program, Georgia Department of Public Health, Atlanta; Division of Infectious Diseases, School of Medicine, Emory University, Atlanta, Georgia; Maryland Department of Health, Baltimore; Michigan Department of Health and Human Services, Lansing; Michigan Department of Health and Human Services, Lansing; Minnesota Department of Health, St Paul; Minnesota Department of Health, St Paul; New Mexico Emerging Infections Program, University of New Mexico, Albuquerque; New Mexico Department of Health, Santa Fe; New York State Department of Health, Albany; New York State Department of Health, Albany; University of Rochester School of Medicine and Dentistry, Rochester, New York; University of Rochester School of Medicine and Dentistry, Rochester, New York; Ohio Department of Health, Columbus; Ohio Department of Health, Columbus; Public Health Division, Oregon Health Authority, Portland; Vanderbilt University Medical Center, Nashville, Tennessee; Vanderbilt University Medical Center, Nashville, Tennessee; Salt Lake County Health Department, Salt Lake City, Utah; Salt Lake County Health Department, Salt Lake City, Utah; Department of Epidemiology, Johns Hopkins Bloomberg School of Public Health, Baltimore, Maryland; Department of Medicine, John Hopkins School of Medicine, Baltimore, Maryland; Influenza Division, Centers for Disease Control and Prevention, Atlanta, Georgia; Influenza Division, Centers for Disease Control and Prevention, Atlanta, Georgia; Influenza Division, Centers for Disease Control and Prevention, Atlanta, Georgia; Connecticut Emerging Infections Program, Yale School of Public Health, New Haven; Influenza Division, Centers for Disease Control and Prevention, Atlanta, Georgia

## Abstract

**IMPORTANCE:**

Seasonal influenza is associated with substantial disease burden. The relationship between census tract–based social vulnerability and clinical outcomes among patients with influenza remains unknown.

**OBJECTIVE:**

To characterize associations between social vulnerability and outcomes among patients hospitalized with influenza and to evaluate seasonal influenza vaccine and influenza antiviral utilization patterns across levels of social vulnerability.

**DESIGN, SETTING, AND PARTICIPANTS:**

This retrospective repeated cross-sectional study was conducted among adults with laboratory-confirmed influenza-associated hospitalizations from the 2014 to 2015 through the 2018 to 2019 influenza seasons. Data were from a population-based surveillance network of counties within 13 states. Data analysis was conducted in December 2023.

**EXPOSURE:**

Census tract–based social vulnerability.

**MAIN OUTCOMES AND MEASURES:**

Associations between census tract–based social vulnerability and influenza outcomes (intensive care unit admission, invasive mechanical ventilation and/or extracorporeal membrane oxygenation support, and 30-day mortality) were estimated using modified Poisson regression as adjusted prevalence ratios. Seasonal influenza vaccine and influenza antiviral utilization were also characterized across levels of social vulnerability.

**RESULTS:**

Among 57 964 sampled cases, the median (IQR) age was 71 (58–82) years; 55.5% (95% CI, 51.5%–56.0%) were female; 5.2% (5.0%–5.4%) were Asian or Pacific Islander, 18.3% (95% CI, 18.0%–18.6%) were Black or African American, and 64.6% (95% CI, 64.2%–65.0%) were White; and 6.6% (95% CI, 6.4%–68%) were Hispanic or Latino and 74.7% (95% CI, 74.3%–75.0%) were non-Hispanic or Latino. High social vulnerability was associated with higher prevalence of invasive mechanical ventilation and/or extracorporeal membrane oxygenation support (931 of 13 563 unweighted cases; adjusted prevalence ratio [aPR], 1.25 [95% CI, 1.13–1.39]), primarily due to socioeconomic status (790 of 11 255; aPR, 1.31 [95% CI, 1.17–1.47]) and household composition and disability (773 of 11 256; aPR, 1.20 [95% CI, 1.09–1.32]). Vaccination status, presence of underlying medical conditions, and respiratory symptoms partially mediated all significant associations. As social vulnerability increased, the proportion of patients receiving seasonal influenza vaccination declined (−19.4% relative change across quartiles; *P* < .001) as did the proportion vaccinated by October 31 (−6.8%; *P* < .001). No differences based on social vulnerability were found in in-hospital antiviral receipt, but early in-hospital antiviral initiation (−1.0%; *P* = .01) and prehospital antiviral receipt (−17.3%; *P* < .001) declined as social vulnerability increased.

**CONCLUSIONS AND RELEVANCE:**

In this cross-sectional study, social vulnerability was associated with a modestly increased prevalence of invasive mechanical ventilation and/or extracorporeal membrane oxygenation support among patients hospitalized with influenza. Contributing factors may have included worsened baseline respiratory health and reduced receipt of influenza prevention and prehospital or early in-hospital treatment interventions among persons residing in low socioeconomic areas.

## Introduction

Seasonal influenza contributes considerably to morbidity and mortality in the United States each year, with an estimated 130 000 to 710 000 hospitalizations and 12 000 to 51 000 deaths annually.^1–3^ Influenza may disproportionately impact socially vulnerable populations, ie, people with community factors that limit disease prevention and management, such as poverty, discrimination, reduced transportation, and dense housing.^4^ Social determinants can contribute to preventable differences in disease burden and health opportunities.^5^ Prior literature highlights disparities in influenza morbidity and mortality based on social characteristics, including race, ethnicity, and poverty.^6–9^ Inadequate reach of evidence-based influenza prevention and control measures may contribute to these disparities.

Seasonal influenza vaccination is the main public health intervention for influenza prevention and disease severity attenuation.^10^ For patients hospitalized with influenza, early antiviral treatment is an additional mitigation measure.^11^ Previous studies show influenza intervention coverage gaps based on factors including race and ethnicity, age, socioeconomic status, education, and area-based social vulnerability.^12–18^ However, understanding how social vulnerability affects influenza outcomes and interventions among hospitalized patients can guide location-specific, context-appropriate health strategy development.

The US Centers for Disease Control and Prevention (CDC) Influenza Hospitalization Surveillance Network (FluSurv-NET) is a population-based system that monitors laboratory-confirmed influenza-associated hospitalizations.^19,20^ Using 2014 to 2015 through 2018 to 2019 season FluSurv-NET data, the primary study objective was to characterize the association between social vulnerability and influenza outcomes among hospitalized patients. The secondary objective was to detect patterns in seasonal influenza vaccination and influenza antiviral utilization among hospitalized patients across levels of social vulnerability.

## Methods

### Design and Setting

A retrospective repeated cross-sectional study was conducted using data from contributing FluSurv-NET sites from 13 US states (California, Colorado, Connecticut, Georgia, Maryland, Michigan, Minnesota, New Mexico, New York, Ohio, Oregon, Tennessee, and Utah; catchment population approximately 9% of US population) during the study period (eTable 1 in Supplement 1). CDC determined that this activity met the requirement for public health surveillance; therefore, CDC Institutional Review Board (IRB) approval was not required. Sites participating in FluSurv-NET obtained approvals from their respective state and local health department and academic partner IRBs as needed. The requirement for informed consent was waived per 45 CFR 46. Study findings are reported following the Strengthening the Reporting of Observational Studies in Epidemiology (STROBE) reporting guideline.

### Study Population

Trained FluSurv-NET site surveillance staff used laboratory, clinical, and notifiable disease databases to identify hospitalized cases and abstract demographic and clinical data using standardized case report forms.^20^ For most cases, race and ethnicity were self-reported, although the source could not be individually confirmed. Race and ethnicity were categorized according to the National Center for Health Statistics (racial groups: American Indian or Alaska Native, Asian or Pacific Islander, Black or African American, multiracial, White, and not specified; ethnic groups: Hispanic or Latino, non-Hispanic or Latino, and not specified). A FluSurv-NET case was defined as a patient who was (1) a surveillance catchment area resident; (2) admitted to the hospital during the influenza season surveillance period (October 1 to April 30); and (3) positive for influenza by a laboratory test (rapid antigen, molecular assay, immunofluorescence assay, or viral culture) within 14 days prior to or anytime during hospitalization. Vaccination status was ascertained from hospital records, state immunization registries, primary care practitioner records, and/or patient or proxy interviews.^20^ A patient was considered to be vaccinated for the influenza season if the vaccine was received starting July 1 and was administered 14 days or more prior to hospitalization. Data capture was comprehensive for most study variables (eTable 2 in Supplement 1). Some FluSurv-NET sites during the 2017 to 2018 and 2018 to 2019 seasons used an age-stratified random sampling scheme to abstract clinical data (eAppendix 1 and eFigure 1 in Supplement 1).^6,21^

### Eligibility

Cases among patients aged at least 18 years were included if residential census tract data was available. Eligibility was restricted to cases presenting with respiratory signs and symptoms (ie, congestion/runny nose, shortness of breath/respiratory distress, cough, sore throat, upper respiratory infections and influenza-like illness [URI/ILI], and/or wheezing occurring within 14 days prior to admission) to control for baseline illness severity. Cases among pregnant or postpartum patients were excluded due to anticipated differences in clinical presentation and admission reasons. Cases with a positive influenza diagnostic test more than 3 days following hospital admission (ie, hospital-onset influenza) were excluded. Cases among patients with a nonresidential address (eg, treatment center, incarcerated persons, nursing facility) were excluded, as were those with an unknown 30-day mortality status.

### Study Outcomes

Primary outcomes were selected to characterize influenza outcomes. They were (1) intensive care unit (ICU) admission; (2) invasive mechanical ventilation (IMV) or extracorporeal membrane oxygenation (ECMO) support; or (3) death during hospitalization or within 30 days following discharge.

Secondary study outcomes characterized influenza vaccination coverage and treatment guideline adherence (eTable 3 in Supplement 1).^11,22^ Two vaccination outcomes were used: proportion receiving seasonal influenza vaccine and proportion vaccinated by the date considered ideal for most people to be vaccinated (October 31).^23^ Three influenza antiviral treatment outcomes were assessed: proportion initiated on recommended in-hospital antivirals, proportion with early (within 1 day following admission) initiation of recommended^11^ in-hospital antivirals, and proportion initiated on antivirals prior to hospital admission.

### Social Vulnerability

The primary exposure was CDC/Agency for Toxic Substances and Disease Registry (ATSDR) Social Vulnerability Index (SVI 2018) ranking.^24^ SVI 2018 applied US census indicators to classify social vulnerability overall and across 4 themes: socioeconomic status (SES), household composition and disabilities, minority status and language, and housing type and transportation (eTable 4 in Supplement 1). A US geographic region relative ranking was assigned ranging from least (0) to most (1) vulnerable. Individual FluSurv-NET sites geocoded patient addresses at admission to identify 2010 census tract, which were matched with SVI 2018 ranking. SVI was stratified into quartiles, with the lowest (Q1, 0–0.25) designated low social vulnerability and the highest (Q4, 0.76–1.0) designated high social vulnerability.

### Statistical Analysis

Demographic and clinical characteristics were reported overall and across SVI quartiles. To account for the complex survey design with stratified sampling, sample weights were used, and variance estimations (95% CIs via 1000 bootstrap replicate weights) were reported. Binary and categorical variables were reported as weighted proportions, and continuous variables as median and IQR. Linear regression was performed to analyze trends across SVI quartiles, with a *P* < .05 significance threshold (2-sided). Relative change in point estimates from lowest (Q1) to highest (Q4) quartile was reported to characterize variation magnitude.

The association between social vulnerability and influenza outcomes was estimated using modified Poisson regression through a generalized linear model with robust error variance.^25–27^ A minimum sufficient model covariates set was derived (eAppendix 2 in Supplement 1), with ICU admission and IMV or ECMO models adjusted for age group (18–49, 50–64, and ≥65 years), number of medical condition categories (0, 1, 2, 3, ≥4), influenza season, sex, and race and ethnicity. Models for death were adjusted for age group, number of medical condition categories, residence type, influenza season, sex, and race and ethnicity. Primary outcome results were reported by SVI quartile (with Q1 as the reference) as adjusted prevalence ratios (aPRs) with 95% CIs, with forest plots produced in Excel for Microsoft 365, version 2402 (Microsoft Corp).

Mediation analysis of influenza vaccination, influenza antiviral receipt, number of medical condition categories, and number of respiratory signs and symptoms was performed for significant prevalence ratios using generalized structural equation modeling.^28–30^ Evidence of mediation was considered when indirect effects were statistically significant, with partial mediation when direct effects were also significant and full mediation when direct effects were not significant.

Two sensitivity analyses were performed. To characterize age as a modifier of the association between SVI and outcomes, results were stratified by age group (18–49 years, 50–64 years, ≥65 years). To explore specific SVI indicators contributing to associations, significant prevalence ratios identified in the primary analysis were further stratified by indicator.

To characterize differences in influenza intervention coverage across SVI quartiles, binary and categorical variables were reported as weighted proportions with 95% CIs. Linear regression was performed to assess trends, with relative change from Q1 to Q4 also reported. Overall SVI results were presented as bar graphs to visually assess trends across quartiles, and descriptive findings were stratified by age groups and theme. Analyses were performed in December 2023 using Stata version 18.0 (StataCorp).

## Results

### Eligibility

Of 82 181 cases among hospitalized influenza patients captured by FluSurv-NET during the 2014 to 2015 through 2018 to 2019 influenza seasons, 20 610 (25.1%) were ineligible for analysis: 8350 (40.5%) had no acute respiratory illness signs or symptoms, 7344 (35.6%) were younger than 18 years, 4902 (23.8%) did not have a residential census tract, and 14 (0.1%) had no overall SVI ranking (eFigure 2 in Supplement 1). Among 61 571 cases, 3607 (5.9%) were excluded: 1344 (37.3%) were among pregnant or postpartum patients, 1314 (36.4%) did not have a residential address, 881 (24.4%) had hospital-onset influenza, and 68 (1.9%) were missing 30-day mortality status. In total, 57 964 cases among hospitalized patients were included.

### Demographic and Clinical Characteristics

Among 57 964 sampled cases, most were patients with influenza type A (81.1% [95% CI, 80.8%–81.5%]), were aged 65 years or older (median [IQR] age, 71 [58–82] years), and were female (55.5% [95% CI, 55.1%–56.0%]). In terms of ethnicity, 6.6% (95% CI, 6.4%–6.8%) were Hispanic or Latino and 74.7% (95% CI, 74.3%–75.0%) were non-Hispanic or Latino. In terms of race, 5.2% (5.0%–5.4%) were Asian or Pacific Islander; 18.3% (95% CI, 18.0%–18.6%), Black or African American; and 64.6% (95% CI, 64.2%–65.0%), White (Table 1). Social vulnerability index ranking was higher for patients who were from the Western census region (relative change from lowest [Q1] to highest [Q4] vulnerability, 12.0%), younger (−12.2%), female sex (6.3%), Hispanic or Latino (339.3%), and a minoritized racial and ethnic group (Black or African American, 504.9%; American Indian or Alaska Native, 166.7%) (all *P* < .001). During the 2017 to 2018 and 2018 to 2019 seasons, the proportion uninsured increased as social vulnerability increased (135.3%; *P* < .001).

Most cases had 1 to 2 respiratory symptoms (59.4% [95% CI, 59.0%–59.8%]), and the median (IQR) number of underlying medical condition categories was 2 (1–3) (Table 2). The number of respiratory signs and symptoms in each patient at admission increased as social vulnerability increased, notably for shortness of breath and respiratory distress (10.6%) and wheezing (20.7%) (both *P* < .001). The proportion of patients with 4 or more categories of medical conditions increased as social vulnerability increased (21.3%), including asthma (60.2%), other chronic lung disease (17.5%), and liver disease (116.1%) (all *P* < .001). Of all cases, 15.0% (95% CI, 14.7%–15.3%) were admitted to the ICU, 5.6% (95% CI, 5.4%–5.8%) received IMV, and 5.3% (95% CI, 5.1–5.4) died in-hospital or within 30 days following discharge. Most deaths occurred among patients aged 65 years or older (80.7% [95% CI, 79.6%–81.8%]). Patients with high social vulnerability had a higher proportion receiving IMV (27.5%; *P* = .03), but no differences were found in ICU admission. Across all ages, the proportion of deaths decreased as social vulnerability increased (−16.7%; *P* < .001). However, for patients in the youngest age group (18–49 years), the proportion of deaths increased as social vulnerability increased (134.3%).

### Prevalence Ratios of Influenza Outcomes

**Figure 1.**
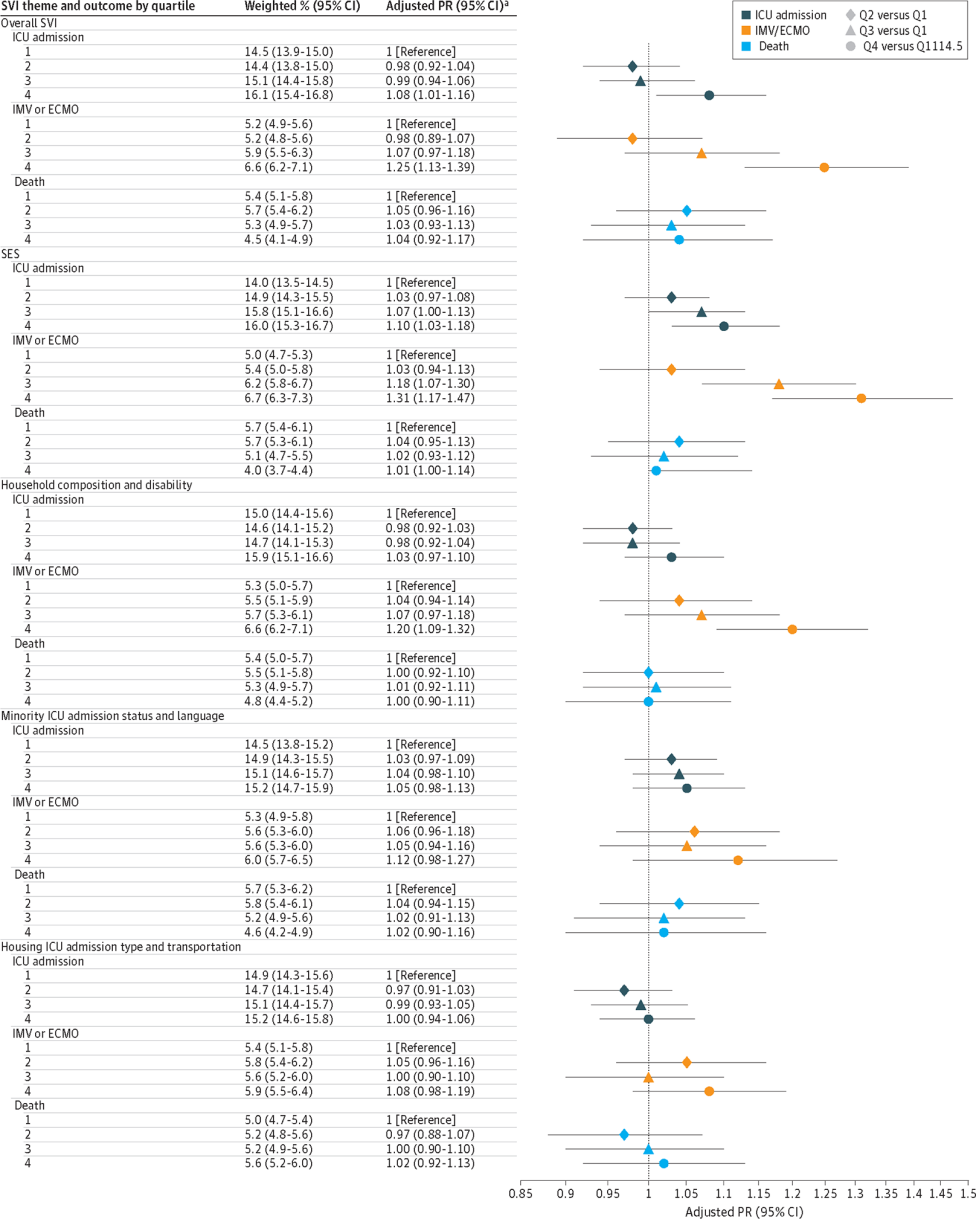
Adjusted Prevalence Ratio (PR) of Influenza Outcomes by Social Vulnerability Index (SVI) 2018 Quartile (Q) in the 2014 to 2015 Through 2018 to 2019 Influenza Seasons, FluSurv-NET Quartile 1 had the lowest vulnerability; and 4, the highest. ^a^ Models for ICU admission and IMV/ECMO adjusted for age, No. of categories of medical conditions, flu season, sex, and race/ethnicity; cases with unknown outcome were excluded. Models for death additionally adjusted for type of residence.

High SVI was associated with increased IMV/ECMO prevalence (Q4, 931 of 13 563 unweighted cases; aPR, 1.25 [95% CI, 1.13–1.39]) (Figure 1). Stratified by theme, these associations were found among those with increased social vulnerability due to low SES (Q4, 790 of 11 255; aPR, 1.31 [95% CI, 1.17–1.47]) and household composition and disability (Q4, 773 of 11 256; aPR, 1.20 [95% CI, 1.09–1.32]). A small association between ICU admission and high SVI was found (Q4, 2226 of 13 556; aPR, 1.08 [95% CI, 1.01–1.16]) and was exclusively based on SES (Q4, 1836 of 11 248; aPR, 1.10 [95% CI, 1.03–1.18]).

Evidence was found of age as an effect modifier between social vulnerability and influenza outcomes (eFigure 3 in Supplement 1). Stratified by age group (18–49, 50–64, and ≥65 years), a higher magnitude of association with IMV/ECMO was found among those aged 65 years or older for both high overall SVI (Q4, 413 of 6278; aPR, 1.50 [95% CI, 1.29–1.76]) and SES (Q4, 319 of 4817; aPR, 1.56 [95% CI, 1.32–1.84]). Associations between ICU admission and high overall SVI and SES were only found among those aged 65 years and older (overall SVI: Q4, 981 of 6275; aPR, 1.18 [95% CI, 1.07–1.30]; SES: 757 of 4816; aPR, 1.21 [95% CI, 1.09–1.34]).

Stratified by SVI indicator, an association was found between ICU admission and the SES income indicator (Q4, 1757 of 10 520; aPR, 1.10 [95% CI, 1.03–1.19]) (eFigure 4 in Supplement 1). Associations with IMV/ECMO were found for all 4 indicators of SES, with the highest for income (Q4, 744 of 10 525; aPR, 1.27 [95% CI, 1.13–1.42]). Associations were only found between IMV/ECMO and household composition and disability indicators of civilians with disability (Q4, 745 of 11 062; aPR, 1.19 [95% CI, 1.07–1.32]) and single-parent household (Q4, 1038 of 15 724; aPR, 1.14 [95% CI, 1.03–1.27]).

Influenza vaccination status was found to partially mediate the association between IMV/ECMO and high SVI (Q4 vs Q1: indirect effect, 0.00 [95% CI, 0.00 to 0.00]; *P* < .001; direct effect, 0.15 [95% CI, 0.05 to 0.24]; *P* < .001) (eTable 5 in Supplement 1). Number of medical condition categories also partially mediated IMV/ECMO and high SVI (Q4 vs Q1: indirect effect, 0.01 [95% CI, 0.01 to 0.02] *P* < .001; direct effect, 0.15 [95% CI, 0.06 to 0.25]; *P* < .001), as did number of respiratory symptoms (Q4 vs Q1: indirect effect, 0.00 [95% CI, −0.01 to 0.00]; *P* < .001; direct effect, 0.19 [95% CI, 0.09 to 0.29]; *P* < .001).

### Influenza Intervention Coverage

**Figure 2.**
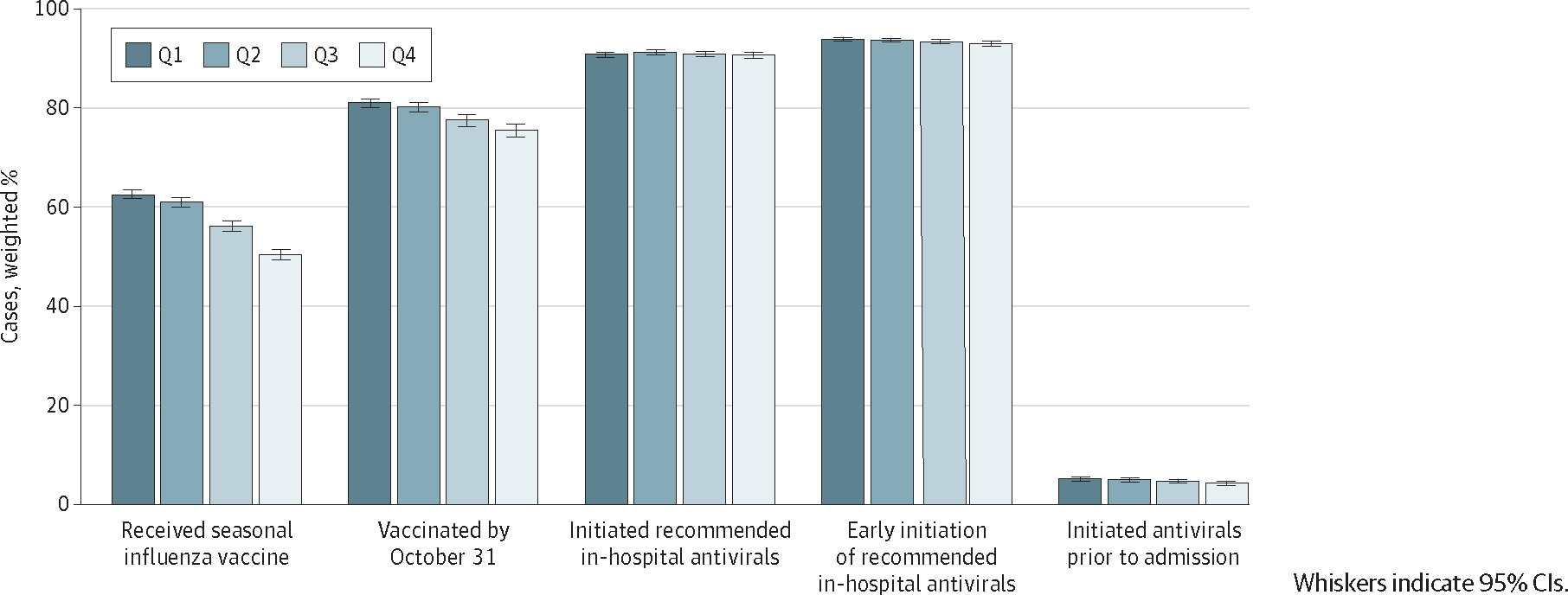
Influenza Intervention Coverage Across Overall Social Vulnerability Index Quartile (Q), 2014 to 2015 Through 2018 to 2019 Influenza Seasons, FluSurv-NET

Of the cases with a known vaccination history, influenza vaccination decreased as social vulnerability increased (−19.4%; *P* < .001) (Figure2 and Table3). This trend was consistent when stratified by age group, with those aged 18 to 49 years having the largest decrease (−17.5%; *P* < .001). Downward trends in proportion vaccinated were consistent across all themes, with SES having the largest decrease (−24.4%; *P* < .001). Among vaccinated patients, the proportion vaccinated by October 31 also decreased as social vulnerability increased (−6.8%; *P* < .001), even when stratified by theme and across most age groups.

No significant difference in in-hospital antiviral receipt was found across overall SVI quartiles (−0.1%; *P* = .66), but a slight decrease was found for SES (−1.1%; *P* = .01) and household composition and disability (−1.1%; *P* = .01). Of the cases initiated on antivirals following admission, a downward trend was detected in early initiation based on overall SVI (−1.0%; *P* = .01), SES (−1.2%; *P* < .001), household composition and disability (−0.6%; *P* = .02), and minority status and language (−0.8%; *P* = .03), but the absolute decrease for all was less than 2%. No trend in early antiviral receipt was found by age group. Finally, the proportion of cases initiated on antivirals prior to admission decreased with higher social vulnerability (−17.3%; *P* < .001), particularly SES (−18.9%; *P* < .001) and housing type and transportation (−13.7%; *P* = .02).

## Discussion

In an analysis of nearly 58 000 cases among patients hospitalized with influenza during the 2014 to 2015 through 2018 to 2019 seasons, high social vulnerability was associated with modestly increased IMV/ECMO prevalence. This association was primarily due to SES and household composition and disability. A smaller association was found between high social vulnerability and ICU admission. Higher IMV/ECMO prevalence was found among increasingly socially vulnerable and older (≥65 years) patients. Among all cases, influenza vaccination receipt, increased number of underlying medical conditions, and increased presence of respiratory symptoms were mediators of the association between social vulnerability and IMV/ECMO. The proportion of cases that were vaccinated declined as overall social vulnerability increased (−19.4%), as did the proportion vaccinated by October 31 (−6.8%). While differences based on social vulnerability were not found for in-hospital influenza antiviral receipt, the proportion of cases initiated on antivirals prior to admission decreased with increasing social vulnerability (−17.3%).

Taken together, these results support that social vulnerability—particularly, living in a low SES census tract— is associated with reduced access to influenza prevention and management measures (ie, seasonal influenza vaccination and prehospital antivirals). Low SES areas also appeared to be associated with general respiratory health, as cases with high social vulnerability had higher proportions of asthma, chronic lung disease, and number of respiratory signs and symptoms. These findings align with prior studies highlighting the relationship between income and smoking, asthma, and chronic obstructive pulmonary disease.^31,32^ Living in lower SES areas can increase exposure to cigarette smoke, environmental pollution, and occupational exposure, and—even controlling for these exposures—directly contributes to decreased lung function.^33^ Influenza illness among cases residing in areas with poorly controlled chronic respiratory diseases and reduced access to earlier medical care may have increased the need for respiratory support once socially vulnerable patients were admitted with influenza.

This is among the first studies to explore social vulnerability among patients hospitalized with influenza. Three COVID-19 studies^34–36^ found an association between social vulnerability (notably SES) and hospital intensive care (organ dysfunction, mechanical ventilation, ICU stay). Prior influenza studies^17,18,37–39^ suggest associations between social vulnerability and decreased influenza vaccination, particularly based on SES. While no study, to our knowledge, has examined in-hospital influenza antiviral use and SVI, a 2021 to 2022 household survey found that those living in more socially vulnerable census tracts were less likely to report receiving antivirals.^40^

### Limitations and Strengths

Several limitations must be considered. First, during the included seasons, FluSurv-NET did not capture individual-level variables that may provide additional context to link SES and IMV/ECMO, including disability status, occupation, income, education, upstream care, and advanced care directives. Second, selection bias may have been introduced as tests captured by FluSurv-NET are completed at clinician discretion or according to hospital policies. While a previous analysis^41^ found that local influenza activity and influenza diagnoses were stronger testing predictors than social determinants, testing may still be influenced by care-seeking behavior, access to care, resource availability, and clinical assessment of illness presentation. Third, eligibility criteria may have limited representativeness to patients with greater access to care. In particular, cases were required to have a geocoded residential census tract, which may exclude those living in rural areas who use post office boxes. Additionally, incarcerated persons and persons experiencing homelessness were excluded due to lack of permanent residential address. Fourth, as FluSurv-NET represents approximately 9% of the US population, findings may not be nationally representative. Fifth, prehospital antivirals underreporting may have occurred due to reliance on hospital medical records.

This analysis has important strengths. First, to our knowledge, this study is the first to examine the link between social vulnerability and influenza outcomes. Identification of disparities in influenza outcomes and interventions can support public health strategy development for socially vulnerable populations. Second, multiseason data provided a larger sample with which to detect associations between social vulnerability, outcomes, and influenza interventions and suggested persistence of these associations across influenza seasons. Third, census tract–level SVI improved geographic granularity on social vulnerability, which can inform disease prevention implementation strategies targeting neighborhoods and communities.

## Conclusions

In this retrospective repeated cross-sectional study of patients hospitalized with influenza during 5 influenza seasons, social vulnerability was associated with a modestly increased IMV/ECMO prevalence. Economic social vulnerability and decreased access to upstream influenza prevention and control interventions including vaccination and prehospital or early in-hospital treatment may have contributed to worsened respiratory health at hospital admission, resulting in increased need for respiratory support. Strategies to prevent severe influenza outcomes should focus on prehospital disease management and attenuation along with improvements in overall respiratory health, particularly for persons living in socioeconomically vulnerable areas.

## Supplementary Material

supp2 SUPPLEMENT 2.
Data Sharing Statement

supp1 SUPPLEMENT 1.
**eAppendix 1.** Sampling Strategy, 2017–2018 and 2018–2019 Seasons
**eAppendix 2.** Primary Outcomes Model Selection
**eTable 1.** FluSurv-NET Counties, 2014–2015 to 2018–2019 Influenza Seasons
**eTable 2.** Missingness Among Key Analytic Variables
**eTable 3.** Secondary Study Outcomes
**eTable 4.** US Centers for Disease Control and Prevention and Agency for Toxic Substances and Disease Registry Social Vulnerability Index 2018 Variables and Source Census Tables
**eTable 5.** Mediation Analysis of Significant Associations Found in Primary Analysis, 2014–2015 to 2018–2019 Influenza Seasons, FluSurv-NET
**eFigure 1.** Random Sampling Scheme for FluSurv-NET 2014–2015 to 2018–2019 Influenza Seasons
**eFigure 2.** Patient Flow Diagram
**eFigure 3.** Adjusted Prevalence Ratio of Influenza Outcomes Stratified by Age Group, 2014–2015 to 2018–2019 Influenza Seasons, FluSurv-NET
**eFigure 4.** Adjusted Prevalence Ratio of Influenza Outcomes by SVI Indicator, 2014–2015 to 2018–2019 Influenza Seasons, FluSurv-NET

## Figures and Tables

**Table 1.** Demographic Characteristics of Cases Among Adult Patients With an Influenza-Associated Hospitalization by Overall SVI 2018 Quartile, 2014 to 2015 Through 2018 to 2019 Influenza Seasons, FluSurv-NET

Characteristic	Weighted column % (95% CI)					*P* value^a^	Relative change, %^b^
	Overall (N = 57 964)	SVI quartile					
		First (low vulnerability) (n = 16 632)	Second (n = 14 966)	Third (n = 12 803)	Fourth (high vulnerability) (N = 13 563)		
SVI quartile							
First	28.6 (28.2 to 29.0)	NA	NA	NA	NA	<.001	−18.9
Second	25.9 (25.5 to 26.3)	NA	NA	NA	NA		
Third	22.3 (22.0 to 22.7)	NA	NA	NA	NA		
Fourth	23.2 (22.8 to 23.5)	NA	NA	NA	NA		
Influenza season							
2014–2015	19.4(19.3 to 19.6)	20.9 (20.4 to 21.4)	20.1 (19.5 to 20.7)	19.0 (18.3 to 19.6)	17.4 (16.9 to 18.0)	<.001	−16.7
2015–2016	8.6 (8.5 to 8.7)	7.8 (7.5 to 8.2)	7.9 (7.6 to 8.3)	8.7 (8.3 to 9.1)	10.2 (9.8 to 10.6)		30.8
2016–2017	19.5 (19.3 to 19.6)	19.5 (19.0 to 20.0)	19.9 (19.3 to 20.5)	19.5 (18.9 to 20.1)	18.9 (18.3 to 19.5)		−3.1
2017–2018	32.7 (32.5 to 32.9)	33.2 (32.4 to 34.0)	32.9 (32.1 to 33.7)	32.8 (31.9 to 33.8)	31.8 (30.9 to 32.6)		−4.2
2018–2019	19.8 (19.7 to 20.0)	18.7 (18.1 to 19.2)	19.2 (18.6 to 19.8)	20.1 (19.5 to 20.8)	21.7 (21.1 to 22.4)		16.0
Month of influenza-associated hospital admission							
October to December	20.8 (20.5 to 21.2)	22.1 (21.4 to 22.8)	20.5 (19.8 to 21.2)	20.9 (20.2 to 21.7)	19.7 (19.0 to 20.4)		−10.9
January to March	71.2 (70.8 to 71.5)	69.7 (68.9 to 70.5)	71.4 (70.6 to 72.1)	71.8 (70.9 to 72.6)	72.1 (71.3 to 72.9)	<.001	3.4
April to June	8.0 (7.8 to 8.2)	8.2 (7.8 to 8.7)	8.1 (7.7 to 8.6)	7.3 (6.9 to 7.8)	8.3 (7.8 to 8.8)		1.2
Influenza type							
Influenza A	81.1 (80.8 to 81.5)	81.3 (80.7 to 81.9)	81.4 (80.7 to 82.1)	81.2 (80.4 to 82.0)	80.5 (79.8 to 81.2)	.14	−1.0
Influenza B	18.4 (18.0 to 18.7)	18.2 (17.6 to 18.8)	18.2 (17.5 to 18.8)	18.3 (17.5 to 19.1)	18.9 (18.2 to 19.6)	.21	3.8
Influenza A and B	0.4 (0.3 to 0.4)	0.3 (0.3 to 0.4)	0.4 (0.3 to 0.5)	0.4 (0.3 to 0.5)	0.5 (0.3 to 0.6)	.18	66.7
Influenza A or B (not distinguishable)	0.1 (0.1 to 0.1)	0.1 (0.1 to 0.2)	0.1 (0.1 to 0.1)	0.1 (0.1 to 0.1)	0.1 (0.1 to 0.2)	.42	0.0
Unknown	0.0 (0.0 to 0.1)	0.0 (0.0 to 0.1)	0.0 (0.0 to 0.1)	0.0 (0.0 to 0.1)	0.1 (0.0 to 0.1)	.09	0.0
Transferred from another hospital	1.3 (1.2 to 1.4)	1.5 (1.3 to 1.7)	1.4 (1.2 to 1.6)	1.3 (1.1 to 1.5)	1.1 (0.9 to 1.3)	.06	−26.7
US Census region^c^							
Midwest	21.8 (21.7 to 22.0)	26.1 (25.5 to 26.8)	20.1 (19.5 to 20.8)	21.5 (20.8 to 22.2)	18.7 (18.1 to 19.3)	<.001	−28.4
Northeast	19.0(18.9 to 19.2)	17.2 (16.7 to 17.8)	23.2 (22.6 to 23.8)	15.0 (14.5 to 15.6)	20.4 (19.8 to 21.0)	.15	18.6
South	24.8 (24.7 to 25.0)	25.1 (24.5 to 25.7)	23.0 (22.4 to 23.7)	26.0 (25.2 to 26.7)	25.5 (24.8 to 26.2)	.02	1.6
West	34.4 (34.2 to 34.5)	31.6 (30.9 to 32.2)	33.7 (33.0 to 34.5)	37.5 (36.7 to 38.4)	35.4 (34.6 to 36.3)	<.001	12.0
Age group, y							
18–49	14.0 (13.9 to 14.2)	10.6 (10.2 to 11.0)	11.9(11.5 to 12.3)	15.1 (14.5 to 15.6)	19.7 (19.1 to 20.3)	<.001	85.8
50–64	23.1 (23.0 to 23.3)	18.6 (18.1 to 19.1)	20.5 (19.9 to 21.1)	24.5 (23.8 to 25.2)	30.3 (29.6 to 31.0)		62.9
≥65	62.9 (62.7 to 63.0)	70.8 (70.2 to 71.4)	67.6 (66.9 to 68.3)	60.5 (59.6 to 61.3)	50.0 (49.2 to 50.9)	−29.4
Age, median (IQR), y	71 (58 to 82)	74 (62 to 84)	73 (60 to 84)	69 (57 to 82)	65 (53 to 77)	<.001	−12.2
Sex							
Female	55.5 (55.1 to 56.0)	53.8 (52.9 to 54.6)	55.4 (54.6 to 56.3)	56.1 (55.2 to 57.1)	57.2 (56.2 to 58.1)	<.001	6.3
Male	44.5 (44.0 to 44.9)	46.2 (45.4 to 47.1)	44.6 (43.7 to 45.4)	43.9 (42.9 to 44.8)	42.8 (41.9 to 43.8)		−7.4
Ethnicity							
Non-Hispanic or Latino	74.7 (74.3 to 75.0)	75.9 (75.3 to 76.6)	75.5 (74.7 to 76.2)	75.4 (74.6 to 76.2)	71.5 (70.7 to 72.3)	<.001	−5.8
Hispanic or Latino	6.6 (6.4 to 6.8)	2.8 (2.5 to 3.0)	4.6 (4.2 to 4.9)	7.9 (7.4 to 8.4)	12.3 (11.7 to 12.9)	<.001	339.3
Not specified	18.8 (18.5 to 19.1)	21.3 (20.7 to 22.0)	20.0 (19.3 to 20.7)	16.7 (16.0 to 17.5)	16.2 (16.0 to 16.9)	<.001	−23.9
Race							
American Indian or Alaska Native	0.5 (0.4 to 0.5)	0.3 (0.3 to 0.4)	0.4 (0.3 to 0.5)	0.5 (0.4 to 0.6)	0.8 (0.6 to 0.9)	<.001	166.7
Asian or Pacific Islander	5.2 (5.0 to 5.4)	3.5 (3.2 to 3.8)	5.9 (5.4 to 6.4)	6.6 (6.1 to 7.1)	5.3 (4.9 to 5.8)	<.001	51.4
Black or African American	18.3 (18.0 to 18.6)	6.1 (5.7 to 6.5)	11.8(11.2 to 12.3)	22.3 (21.5 to 23.1)	36.9 (36.1 to 37.7)	<.001	504.9
Multiracial	0.3 (0.2 to 0.3)	0.2 (0.2 to 0.3)	0.2 (0.1 to 0.3)	0.4 (0.3 to 0.5)	0.3 (0.2 to 0.5)	.03	50.0
White	64.6 (64.2 to 65.0)	80.5 (79.9 to 81.2)	71.3 (70.5 to 72.0)	59.3 (58.4 to 60.2)	42.7 (41.8 to 43.6)	<.001	−47.0
Not specified	11.1 (10.9 to 11.4)	9.4 (8.9 to 9.8)	10.5 (10.0 to 11.1)	11.0 (10.5 to 11.6)	14.0 (13.4 to 14.6)	<.001	48.9
Insurance status^d^							
Uninsured	2.8 (2.6 to 3.0)	1.7 (1.5 to 2.0)	2.4 (2.1 to 2.7)	3.2 (2.8 to 3.7)	4.0 (3.6 to 4.5)	<.001	135.3
Insured	97.2 (97.0 to 97.4)	98.3 (98.0 to 98.6)	97.6 (97.3 to 97.9)	96.8 (96.3 to 97.2)	96.0 (95.5 to 96.4)		−2.3
Private	49.4 (48.8 to 50.1)	56.3 (55.0 to 57.6)	54.2 (52.7 to 55.6)	46.4 (44.9 to 47.9)	38.6 (37.2 to 40.0)	<.001	−31.4
Medicare	65.4 (64.9 to 66.0)	68.5 (67.4 to 69.6)	68.8 (67.5 to 70.0)	64.6 (63.3 to 65.9)	58.7 (57.3 to 60.1)	<.001	−14.3
Medicaid or state assistance	25.7 (25.2 to 26.3)	12.8 (12.0 to 13.6)	21.3 (20.2 to 22.4)	29.2 (27.9 to 30.5)	43.4 (42.1 to 44.8)	<.001	239.1
Military	3.3 (3.0 to 3.6)	3.6 (3.1 to 4.2)	3.0 (2.5 to 3.5)	3.7 (3.2 to 4.4)	2.8 (2.3 to 3.3)	.12	−22.2
Indian Health Service	0.1 (0.0 to 0.1)	0.0 (0.0 to 0.1)	0.0(0.0to0.0)	0.1 (0.0 to 0.2)	0.1 (0.0 to 0.3)	.14	0.0
Other	1.3 (1.1 to 1.5)	1.5 (1.1 to 1.9)	1.0 (0.7 to 1.4)	1.3 (1.0 to 1.7)	1.4 (1.1 to 1.8)	.97	−6.7

Abbreviations: NA, not applicable; SVI, Social Vulnerability Index.

a Linear regression was used to analyze trends across SVI quartiles.

b Relative change was calculated as the weighted proportion or median value of quartile 4 (high vulnerability) minus quartile 1 (low vulnerability), divided by the weighted proportion or median value of quartile 1 (low vulnerability).

c US Census regions include Midwest (Michigan, Minnesota, and Ohio), Northeast (Connecticut and New York), South (Georgia, Maryland, and Tennessee), andWest (California, Colorado, New Mexico, Oregon, and Utah).

d Insurance status only collected by FluSurv-NET for the 2017 to 2018 and 2018 to 2019 influenza seasons. Type of insurance is not mutually exclusive.

**Table 2.** Clinical Characteristics of Cases Among Adult Patients With an Influenza-Associated Hospitalization by Overall SVI 2018 Quartile, 2014 to 2015 Through 2018 to 2019 Influenza Seasons, FluSurv-NET

Characteristic	Weighted column, % (95% CI)					*P* value^a^	Relative change, %^b^
	Overall (n = 57 964)	SVI quartile					
		First (low vulnerability) (n = 16 632)	Second (n = 14 966)	Third (n = 12 803)	Fourth (high vulnerability) (n = 13 563)		
No. of respiratory signs and symptoms, median (IQR)							
Overall	2 (2–3)	2 (1–3)	2 (2–3)	2 (2–3)	2 (2–3)	<.001	0
18–49 y	2 (2–3)	2 (2–3)	2 (2–3)	2 (2–3)	2 (2–3)	.001	0
50–64 y	2 (2–3)	2 (2–3)	2 (2–3)	2 (2–3)	2 (2–3)	<.001	0
≥65 y	2 (1–3)	2 (1–3)	2 (1–3)	2 (1–3)	2 (1–3)	.14	0
No. of respiratory signs/symptoms							
1–2	59.4 (59.0–59.8)	61.0 (60.2–61.8)	60.2 (59.3–61.0)	59.5 (58.5–60.4)	56.5 (55.6–57.4)	<.001	−7.4
3–4	37.0 (36.6–37.4)	35.6 (34.8–36.4)	36.4 (35.6–37.3)	37.0 (36.1–37.9)	39.2 (38.3–40.1)		10.1
5–6	3.7 (3.5–3.8)	3.4 (3.1–3.7)	3.4 (3.1–3.7)	3.6 (3.3–4.0)	4.3 (3.9–4.6)		26.5
Signs and symptoms at admission							
Respiratory							
Congested/runny nose	29.8 (29.4–30.2)	30.3 (29.6–31.1)	30.0 (29.2–30.8)	28.9 (28.1–29.8)	29.9 (29.1–30.7)	.17	−1.3
Cough	89.2 (88.9–89.4)	89.6 (89.1–90.1)	89.2 (88.6–89.7)	89.1 (88.5–89.7)	88.6 (88.0–89.2)	.02	−1.1
Shortness of breath or respiratory distress	64.8 (64.4–65.2)	62.2 (61.4–63.0)	63.3 (62.4–64.2)	65.7 (64.8–66.6)	68.8 (67.9–69.6)	<.001	10.6
Sore throat	14.6 (14.3–14.9)	14.6 (14.0–15.2)	13.8 (13.3–14.4)	13.9 (13.3–14.6)	16.1 (15.4–16.7)	<.001	10.3
URI/ILI^c^	12.6 (12.3–12.9)	10.2 (9.7–10.7)	9.9 (9.4–10.4)	10.7 (10.1–11.3)	9.8 (9.3–10.4)	.15	−3.9
Wheezing	26.7 (26.3–27.1)	24.2 (23.5–25.0)	27.2 (26.4–28.0)	26.8 (26.0–27.7)	29.2 (28.4–30.0)	<.001	20.7
Nonrespiratory							
Altered mentalstatus or confusion	14.5 (14.2–14.8)	15.8 (15.2–16.4)	15.9 (15.3–16.6)	14.3 (13.6–15.0)	11.4 (10.8–12.0)	<.001	−27.8
Fever or chills	65.0 (64.5–65.4)	64.6 (63.7–65.4)	64.0 (63.2–64.9)	64.6 (63.7–65.5)	66.9 (66.0–67.7)	<.001	3.6
Seizures	0.4 (0.4–0.5)	0.4 (0.3–0.5)	0.3 (0.3–0.5)	0.4 (0.3–0.6)	0.5 (0.4–0.6)	.28	25.0
Smoking status (tobacco)							
Current	19.0 (18.7–19.4)	13.6 (13.1–14.2)	16.8 (16.2–17.4)	20.4 (19.6–21.1)	26.9 (26.1–27.7)	<.001	97.8
Former	33.2 (32.8–33.7)	35.8 (35.0–36.7)	35.5 (34.6–36.4)	31.7 (30.8–32.5)	29.0 (28.1–29.9)	<.001	−19.0
No or unknown	47.8 (47.3–48.2)	50.6 (49.7–51.4)	47.7 (46.8–48.6)	48.0 (47.0–49.0)	44.1 (43.2–45.0)	<.001	−12.8
Alcohol use disorder							
Current	2.8 (2.7–3.0)	2.4 (2.1–2.6)	2.6 (2.4–2.9)	2.7 (2.4–3.0)	3.8 (3.5–4.2)	<.001	58.3
Former	2.8 (2.7–3.0)	2.1 (1.9–2.3)	2.4 (2.2–2.7)	3.0 (2.7–3.3)	3.9 (3.6–4.3)	<.001	85.7
No or unknown	94.4 (94.2–94.6)	95.6 (95.2–95.9)	95.0 (94.6–95.3)	94.3 (93.9–94.7)	92.3 (91.8–92.7)	<.001	−3.5
BMI, median (IQR)	27.7 (23.5–33.3)	27.1 (23.3–32.1)	27.4 (23.5–32.9)	28.0 (23.7–33.8)	28.6 (23.8–34.8)	<.001	5.5
BMI category							
Underweight (<18.5)	4.0 (3.9–4.2)	3.8 (3.5–4.2)	4.2 (3.8–4.6)	4.1 (3.7–4.4)	4.1 (3.8–4.5)	.01	7.9
Normalor healthy weight (18.5–24.9)	27.2 (26.8–27.6)	29.8 (29.0–30.6)	28.0 (27.2–28.8)	25.8 (25.0–26.7)	24.4 (23.5–25.2)		−18.1
Overweight (25.0–29.9)	26.9 (26.5–27.3)	28.3 (27.5–29.0)	27.7 (27.0–28.5)	26.7 (25.8–27.6)	24.3 (23.6–25.1)		−14.1
Obesity (30.0–39.9)	26.5 (26.1–26.9)	25.3 (24.6–26.1)	25.5 (24.7–26.3)	27.4 (26.5–28.2)	28.3 (27.5–29.1)		11.9
Morbid obesity (≥40.0)	9.8 (9.5–10.1)	7.3 (6.9–7.7)	9.2 (8.7–9.7)	10.6 (10.1–11.1)	12.7 (12.2–13.3)		74.0
Unknown	5.6 (5.4–5.8)	5.6 (5.2–6.0)	5.4 (5.0–5.8)	5.4 (5.0–5.9)	6.2 (5.7–6.6)		10.7
Influenza-associated pneumonia^d^	23.3 (22.9–23.7)	24.1 (23.4–24.9)	22.3 (21.5–23.0)	23.3 (22.5–24.1)	23.5 (22.8–24.3)	.53	−2.5
No. of categories of medical conditions^e^							
0	9.5 (9.3–9.8)	10.3 (9.8–10.8)	9.1 (8.6–9.6)	9.3 (8.7–9.8)	9.3 (8.8–9.8)	<.001	−9.7
1	23.6 (23.2–23.9)	24.7 (24.0–25.4)	23.5 (22.7–24.2)	23.1 (22.4–24.0)	22.7 (21.9–23.4)		−8.1
2	27.7 (27.3–28.1)	28.4 (27.7–29.2)	28.0 (27.2–28.8)	27.6 (26.7–28.5)	26.7 (25.9–27.5)		−6.0
3	21.5 (21.1–21.9)	20.7 (20.0–21.4)	21.5 (20.8–22.3)	21.9 (21.1–22.7)	21.9 (21.2–22.7)		5.8
≥4	17.8 (17.4–18.1)	16.0 (15.4–16.6)	18.0 (17.3–18.6)	18.1 (17.4–18.8)	19.4(18.7–20.2)		21.3
No. of categories of medical conditions, median (IQR)^e^							
Overall	2 (1–3)	2 (1–3)	2 (1–3)	2 (1–3)	2 (1–3)	<.001	0
18–49 y	2 (1–2)	2 (1–2)	2 (1–2)	2 (1–2)	2 (1–2)	<.001	0
50–64 y	2 (1–3)	2 (1–3)	2 (1–3)	2 (1–3)	2 (1–3)	<.001	0
≥65 y	2 (1–3)	2 (1–3)	2 (1–3)	2 (1–3)	2 (2–3)	<.001	0
Type of category of medical condition							
Asthma	20.6 (20.3–21.0)	16.6 (16.0–17.2)	19.2 (18.5–19.8)	21.3 (20.6–22.1)	26.6 (25.8–27.4)	<.001	60.2
Chronic lung disease	31.8 (31.4–32.2)	29.1 (28.3–30.0)	32.3 (31.5–33.1)	32.3 (31.4–33.2)	34.2 (33.3–35.1)	<.001	17.5
Chronic metabolic disease	45.1 (44.7–45.6)	43.4 (42.6–44.2)	45.2 (44.3–46.1)	46.2 (45.2–47.1)	46.3 (45.3–47.2)	<.001	6.7
Blood disorders or hemoglobinopathy	4.0 (3.9–4.2)	3.9 (3.6–4.2)	4.1 (3.8–4.4)	3.9 (3.5–4.3)	4.3 (4.0–4.7)	.13	10.3
Cardiovascular disease	51.1 (50.6–51.5)	52.4 (51.6–53.3)	53.4 (52.5–54.3)	50.7 (49.8–51.6)	47.1 (46.2–48.0)	<.001	−10.1
Neuromuscular disorder	5.8 (5.6–6.0)	6.7 (6.3–7.1)	5.8 (5.4–6.3)	5.4 (5.0–5.9)	5.1 (4.8–5.6)	<.001	−23.9
Neurologic disorder	20.3 (20.0–20.7)	20.8 (20.1–21.4)	21.0 (20.3–21.7)	21.0 (20.2–21.8)	18.4 (17.7–19.1)	<.001	−11.5
History of Guillain-Barré syndrome	0.1 (0.1–0.2)	0.2 (0.1–0.3)	0.2 (0.1–0.2)	0.1 (0.1–0.2)	0.1 (0.0–0.1)	<.001	−50.0
Immunocompromising condition	18.0 (17.6–18.3)	18.67 (18.0–19.3)	17.6 (17.0–18.3)	17.1 (16.4–17.8)	18.3 (17.6–19.0)	.23	−2.0
Kidney disease	21.6 (21.2–21.9)	20.1 (19.4–20.8)	21.5 (20.8–22.3)	22.7 (21.9–23.5)	22.3 (21.6–23.1)	<.001	10.9
Liver disease	4.3 (4.1–4.4)	3.1 (2.8–3.4)	3.4 (3.1–3.7)	4.3 (3.9–4.7)	6.7 (6.3–7.2)	<.001	116.1
ICU admission							
No	85.0 (84.7–85.3)	85.5 (84.9–86.0)	85.5 (84.9–86.1)	84.9 (84.2–85.5)	83.9 (83.1–84.5)	.72	−1.9
Yes	15.0 (14.7–15.3)	14.5 (13.9–15.0)	14.4 (13.8–15.0)	15.1 (14.4–15.8)	16.1 (15.4–16.8)		11.0
Unknown	0.1 (0.0–0.1)	0.1 (0.0–0.1)	0.1 (0.0–0.1)	0.1 (0.0–0.1)	0.1 (0.0–0.1)		0.0
Invasive mechanical ventilation							
No	94.3 (94.1–94.5)	94.8 (94.5–95.2)	94.8 (94.4–95.2)	94.2 (93.7–94.5)	93.3 (92.9–93.8)	.03	−1.6
Yes	5.6 (5.4–5.8)	5.1 (4.8–5.4)	5.1 (4.7–5.5)	5.7 (5.4–6.1)	6.5 (6.1–7.0)		27.5
Unknown	0.1 (0.1–0.1)	0.1 (0.1–0.1)	0.1 (0.1–0.2)	0.1 (0.1–0.2)	0.2 (0.1–0.2)		100.0
Extracorporeal membrane oxygenation							
No	99.6 (99.5–99.6)	99.6 (99.5–99.7)	99.6 (99.4–99.7)	99.6 (99.4–99.7)	99.6 (99.5–99.7)	.23	0.0
Yes	0.3 (0.2–0.3)	0.3 (0.2–0.4)	0.3 (0.2–0.4)	0.3 (0.2–0.4)	0.2 (0.2–0.4)		−33.3
Unknown	0.2 (0.1–0.2)	0.1 (0.1–0.2)	0.2 (0.1–0.3)	0.1 (0.1–0.2)	0.2 (0.1–0.3)		100.0
Died^f^							
No	94.7 (94.6–94.9)	94.6 (94.2–94.9)	94.3 (93.9–94.7)	94.7 (94.3–95.1)	95.5 (95.1–95.9)	<.001	1.0
Yes	5.3 (5.1–5.4)	5.4 (5.1–5.8)	5.7 (5.4–6.2)	5.3 (4.9–5.7)	4.5 (4.1–4.9)		−16.7
18–49 y	5.0 (4.5–5.7)	3.5 (2.6–4.8)	4.6 (3.5–6.0)	4.8 (3.6–6.2)	8.2 (6.5–10.2)	<.001	134.3
50–64 y	14.3 (13.3–15.3)	11.6 (10.0–13.4)	11.1 (9.4–13.0)	17.6 (15.2–20.3)	19.1 (16.4–22.1)		64.7
≥65 y	80.7 (79.6–81.8)	84.9 (82.8–86.7)	84.3 (82.1–86.3)	77.7 (74.8–80.3)	72.8 (69.5–75.8)		−14.3

Abbreviations: BMI, body mass index (calculated as weight in kilograms divided by height in meters squared); ICU, intensive care unit; SVI, Social Vulnerability Index; URI/ILI, upper respiratory infections and influenza-like illness.

a Linear regression was used to analyze trends across SVI quartiles.

b Relative change was calculated as the weighted proportion or median value of quartile 4 (high vulnerability) minus quartile 1 (low vulnerability) divided by the weighted proportion or median value of quartile 1 (low vulnerability).

c URI/ILI was not collected during the 2014 to 2015 influenza season.

d Influenza-associated pneumonia includes abnormal chest radiograph findings and diagnostic codes indicating pneumonia, including *International Classification of Diseases, Ninth Revision* codes 480, 481, 482, 483, 484, 485, 486, 487, 510, 513, and 997.31 and *International Statistical Classification of Diseases and Related Health Problems, Tenth Revision* codes J09, J10, J11, J12, J13, J14, J15, J16, J17, J18, J85.1, J86.9, and J95.851.

e Categories of medical conditions include asthma, chronic lung disease, chronic metabolic disease, blood disorders or hemoglobinopathy, cardiovascular disease, neuromuscular disorder, neurologic disorder, history of Guillain-Barré Syndrome, immunocompromised condition, kidney disease, and liver disease.

f Includes both deaths occurring in-hospital and within 30 days of hospital discharge. Patients with unknown final outcomes of alive or deceased at the time of hospital discharge were excluded from the analysis.

**Table 3.** Influenza Intervention Coverage by SVI 2018 Quartile, 2014 to 2015 Through 2018 to 2019 Influenza Seasons, FluSurv-NET

Intervention	Weighted % (95% CI)					*P* value^a^	Relative change, %^b^
	Overall	SVI quartile					
		First (low vulnerability)	Second	Third	Fourth (high vulnerability)		
**Received seasonal influenza vaccine**							
Overall SVI	58.0 (57.5–58.4)	62.5 (61.7–63.4)	61.0 (60.1–61.9)	56.2 (55.2–57.2)	50.4(49.4–51.4)	<.001	−19.4
18–49 y	32.9 (31.8–33.9)	37.2 (35.0–39.5)	33.8 (31.6–36.1)	31.0 (28.9–33.2)	30.7 (28.9–32.5)	<.001	−17.5
50–64 y	46.9 (46.0–47.8)	48.6 (46.8–50.4)	48.0 (46.1–49.9)	46.5 (44.6–48.5)	45.1 (43.5–46.7)	<.001	−7.2
≥65 y	67.5 (66.9–68.1)	69.9 (68.9–70.9)	69.5 (68.4–70.7)	66.3 (65.0–67.5)	61.3 (59.8–62.9)	<.001	−12.3
SVI theme 1: SES	NA	64.4 (63.6–65.2)	59.4 (58.5–60.3)	54.1 (53.0–55.1)	48.7 (47.6–49.7)	<.001	−24.4
SVI theme 2: household composition and disability	NA	59.8 (58.9–60.7)	59.8 (58.9–60.7)	57.1 (56.2–58.1)	53.4 (52.3–54.5)	<.001	−10.7
SVI theme 3: minority status and language	NA	62.5 (61.4–63.6)	61.7 (60.8–62.5)	57.5 (56.7–58.4)	51.9 (51.0–52.9)	<.001	−17.0
SVI theme 4: housing type and transportation	NA	58.7 (57.7–59.6)	58.3 (57.3–59.2)	58.8 (57.9–59.7)	56.2 (55.3–57.1)	<.001	−4.3
**Proportion vaccinated by October 31**							
Overall SVI	78.9 (78.4–79.4)	81.0 (80.1–81.8)	80.2 (79.2–81.1)	77.6 (76.3–78.8)	75.5 (74.3–76.7)	<.001	−6.8
18–49 y	71.7 (69.9–73.4)	74.3 (70.7–77.7)	71.5 (67.7–75.0)	71.7 (67.9–75.2)	69.7 (66.3–72.8)	.07	−6.2
50–64 y	75.0 (73.8–76.1)	77.4 (75.2–79.5)	75.1 (72.7–77.3)	74.6 (72.1–77.0)	73.2 (70.9–75.4)	.01	−5.4
≥65 y	80.7 (80.1–81.3)	82.1 (81.1–83.1)	82.0 (80.8–83.1)	79.1 (77.5–80.5)	77.7 (76.1–79.3)	<.001	−5.4
SVI theme 1: SES	NA	81.8 (81.0–82.6)	78.7 (77.7–79.7)	77.6 (76.3–78.8)	74.1 (72.7–75.4)	<.001	−9.4
SVI theme 2: household composition and disability	NA	80.2 (79.3–81.1)	79.6 (78.6–80.5)	78.7 (77.7–79.7)	76.0 (74.6–77.3)	<.001	−5.2
SVI theme 3: minoritystatus and language	NA	80.8 (79.7–81.9)	80.1 (79.1–81.1)	79.0 (78.0–80.0)	76.0 (74.9–77.1)	<.001	−5.9
SVI theme 4: housing type and transportation	NA	79.8 (78.9–80.8)	79.1 (78.0–80.1)	79.6 (78.5–80.7)	77.3 (76.2–78.3)	<.001	−3.1
**Initiated on recommended in-hospital antivirals** ^c^							
Overall SVI	90.9 (90.7–91.1)	90.8 (90.3–91.2)	91.3 (90.8–91.7)	90.9 (90.4–91.5)	90.7 (90.1–91.2)	.66	−0.1
18–49 y	89.5 (88.8–90.1)	88.1 (86.6–89.5)	89.4 (87.9–90.7)	90.0 (88.6–91.2)	90.1 (88.9–91.1)	.04	2.3
50–64 y	89.7 (89.2–90.2)	88.8 (87.7–89.9)	90.5 (89.5–91.5)	89.7 (88.6–90.7)	89.8 (88.9–90.7)	.41	1.1
≥65 y	91.7 (91.4–92.0)	91.7 (91.1–92.2)	91.8 (91.2–92.4)	91.7 (91.0–92.4)	91.4 (90.6–92.1)	.63	−0.3
SVI theme 1: SES	NA	91.3 (90.8–91.8)	91.0 (90.5–91.5)	90.8 (90.2–91.3)	90.3 (89.7–90.9)	.01	−1.1
SVI theme 2: household composition and disability	NA	91.3 (90.8–91.8)	91.0 (90.5–91.5)	90.8 (90.2–91.3)	90.3 (89.7–90.9)	.01	−1.1
SVI theme 3: minority status and language	NA	89.9 (89.3–90.5)	90.6 (90.1–91.1)	91.0 (90.5–91.4)	91.8 (91.3–92.2)	<.001	2.1
SVI theme 4: housing type and transportation	NA	90.7 (90.2–91.2)	91.1 (90.6–91.6)	90.9 (90.5–91.4)	90.8 (90.3–91.3)	.88	0.1
**Early initiation of recommended in-hospital antivirals** ^c^							
Overall SVI	93.5 (93.3–93.7)	93.9 (93.4–94.3)	93.7 (93.2–94.2)	93.4 (92.9–93.9)	93.0 (92.5–93.5)	.01	−1.0
18–49 y	92.7 (92.2–93.3)	92.7 (91.3–93.8)	93.3 (92.0–94.3)	92.4 (91.1–93.5)	92.7 (91.7–93.6)	.77	0.0
50–64 y	92.8 (92.3–93.2)	93.0 (91.9–93.9)	93.0 (91.9–94.0)	92.8 (91.7–93.7)	92.4(91.5–93.2)	.37	−0.6
≥65 y	94.0 (93.7–94.3)	94.3 (93.7–94.8)	94.0 (93.3–94.6)	93.9 (93.2–94.5)	93.5 (92.7–94.1)	.08	−0.8
SVI theme 1: SES	NA	93.9 (93.5–94.3)	93.7 (93.2–94.2)	93.3 (92.8–93.8)	92.8 (92.2–93.3)	<.001	−1.2
SVI theme 2: household composition and disability	NA	93.7 (93.2–94.1)	94.0 (93.5–94.4)	93.1 (92.6–93.6)	93.1 (92.5–93.6)	.02	−0.6
SVI theme 3: minority status and language	NA	94.2 (93.7–94.7)	93.5 (93.1–94.0)	93.2 (92.8–93.7)	93.4 (93.0–93.8)	.03	−0.8
SVI theme 4: housing type and transportation	NA	93.6 (93.2–94.1)	93.6 (93.1–94.1)	93.3 (92.8–93.8)	93.6 (93.1–94.0)	.65	0.0
**Initiated on recommended antivirals prior to admission** ^c^							
Overall SVI	4.8 (4.6–5.1)	5.2 (4.8–5.6)	5.0 (4.6–5.4)	4.7 (4.3–5.1)	4.3 (3.9–4.8)	<.001	−17.3
18–49 y	5.1 (4.6–5.6)	6.0 (5.1–7.2)	5.3 (4.4–6.5)	4.7 (3.9–5.7)	4.5 (3.8–5.4)	.02	−25.0
50–64 y	4.6 (4.2–5.0)	4.7 (4.0–6.0)	5.1 (4.4–6.0)	4.3 (3.6–5.1)	4.4 (3.7–5.1)	.27	−6.4
≥65 y	4.9 (4.6–5.2)	5.2 (4.7–5.7)	4.9 (4.4–5.4)	4.8 (4.3–5.5)	4.2 (3.7–4.9)	.03	−19.2
SVI theme 1: SES	NA	5.3 (4.9–5.7)	4.7 (4.4–5.2)	4.7 (4.3–5.1)	4.3 (3.9–4.7)	<.001	−18.9
SVI theme 2: household composition and disability	NA	5.1 (4.7–5.6)	4.8 (4.5–5.3)	4.5 (4.1–4.9)	4.7 (4.3–5.2)	.08	−7.8
SVI theme 3: minority status and language	NA	4.9 (4.4–5.4)	5.0 (4.6–5.5)	4.9 (4.5–5.3)	4.6 (4.2–5.0)	.24	−6.1
SVI theme 4: housing type and transportation	NA	5.1 (4.7–5.5)	5.0 (4.6–5.4)	4.8 (4.4–5.3)	4.4 (4.0–4.8)	.02	−13.7

Abbreviations: NA, not applicable; SES, socioeconomic status; SVI, Social Vulnerability Index.

a Linear regression performed to evaluate trend across SVI quartiles.

b Relative change was calculated as the weighted proportion or median value of quartile 4 (high vulnerability) minus quartile 1 (low vulnerability), divided by the weighted proportion or median value of quartile 1 (low vulnerability).

c Includes neuraminidase inhibitors (oseltamivir, peramivir, and zanamivir) and baloxavir marboxil.

## Data Availability

See Supplement 2.

## Key Points

### Question

Is social vulnerability associated with outcomes and interventions among patients hospitalized with influenza?

### Findings

In this cross-sectional study of 57 964 sampled cases, high census tract–based social vulnerability was associated with increased prevalence of respiratory support compared with low vulnerability, most notably for cases among patients living in low socioeconomic areas.

### Meaning

These findings suggest that persons residing in socially vulnerable, lower-income areas may experience worsened respiratory health, which could contribute to increased need for respiratory support once hospitalized with influenza.
