# Supplementary material for: Social Vulnerability, Intervention Utilization, and Outcomes in US Adults Hospitalized With Influenza
Source: JAMA Netw Open. Author manuscript; Available in PMC 2025 Jan 6. (PMC11702903; doi:10.1001/jamanetworkopen.2024.48003)
Supplement: supp2 — SUPPLEMENT 2. Data Sharing Statement [file NIHMS2045202-supplement-supp2.pdf]

Data Sharing Statement

Adams. Social Vulnerability, Intervention Utilization, and Outcomes in US Adults Hospitalized With Influenza. *JAMA Netw Open*. Published November 27, 2024.
doi:10.1001/jamanetworkopen.2024.48003

Data
Data available: No
