# Supplementary material for: Social Vulnerability, Intervention Utilization, and Outcomes in US Adults Hospitalized With Influenza
Source: JAMA Netw Open. Author manuscript; Available in PMC 2025 Jan 6. (PMC11702903; doi:10.1001/jamanetworkopen.2024.48003)
Supplement: supp1 — SUPPLEMENT 1. eAppendix 1. Sampling Strategy, 2017–2018 and 2018–2019 Seasons eAppendix 2. Primary Outcomes Model Selection eTable 1. FluSurv-NET Counties, 2014–2015 to 2018–2019 Influenza Seasons eTable 2. Missingness Among Key Analytic Variables eTable 3. Secondary Study Outcomes eTable 4. US Centers for Disease Control and Prevention and Agency for Toxic Substances and Disease Registry Social Vulnerability Index 2018 Variables and Source Census Tables eTable 5. Mediation Analysis of Significant Associations Found in Primary Analysis, 2014–2015 to 2018–2019 Influenza Seasons, FluSurv-NET eFigure 1. Random Sampling Scheme for FluSurv-NET 2014–2015 to 2018–2019 Influenza Seasons eFigure 2. Patient Flow Diagram eFigure 3. Adjusted Prevalence Ratio of Influenza Outcomes Stratified by Age Group, 2014–2015 to 2018–2019 Influenza Seasons, FluSurv-NET eFigure 4. Adjusted Prevalence Ratio of Influenza Outcomes by SVI Indicator, 2014–2015 to 2018–2019 Influenza Seasons, FluSurv-NET [file NIHMS2045202-supplement-supp1.pdf]

# Supplemental Online Content

Adams K, Yousey-Hindes K, Bozio CH, et al. Social vulnerability, intervention utilization, and outcomes in US adults hospitalized with influenza. *JAMA Netw Open.* 2024;7(11):e2448003. doi:10.1001/jamanetworkopen.2024.48003

**eAppendix 1.** Sampling Strategy, 2017-2018 and 2018-2019 Seasons

**eAppendix 2.** Primary Outcomes Model Selection

**eTable 1.** FluSurv-NET Counties, 2014-2015 to 2018-2019 Influenza Seasons

**eTable 2.** Missingness Among Key Analytic Variables

**eTable 3.** Secondary Study Outcomes

**eTable 4.** US Centers for Disease Control and Prevention and Agency for Toxic Substances and Disease Registry Social Vulnerability Index 2018 Variables and Source Census Tables

**eTable 5.** Mediation Analysis of Significant Associations Found in Primary Analysis, 2014-2015 to 2018-2019 Influenza Seasons, FluSurv-NET

**eFigure 1.** Random Sampling Scheme for FluSurv-NET 2014-2015 to 2018-2019 Influenza Seasons

**eFigure 2.** Patient Flow Diagram

**eFigure 3.** Adjusted Prevalence Ratio of Influenza Outcomes Stratified by Age Group, 2014-2015 to 2018-2019 Influenza Seasons, FluSurv-NET

**eFigure 4.** Adjusted Prevalence Ratio of Influenza Outcomes by SVI Indicator, 2014-2015 to 2018-2019 Influenza Seasons, FluSurv-NET

This supplemental material has been provided by the authors to give readers additional information about their work.

## eAppendix 1. Sampling Strategy, 2017-2018 and 2018-2019 Seasons

Due to the high volume of influenza-associated hospitalizations among older-aged adults, some FluSurv-NET sites during the 2017–2018 and 2018–2019 seasons used a random stratified sampling scheme to abstract clinical data by age strata. Briefly, a random number was assigned to each case following FluSurv-NET database entry. At sites using random sampling, complete clinical data was abstracted for all patients who died within 30 days of hospitalization. Among patients who survived, stratification by age group was performed to select a representative sample among older-aged adults. During the 2017–2018 season, six sites abstracted 50% of cases among patients aged 50–64 years, and 25% among patients aged ≥65 years. One site during the 2017–2018 season and all seven sites that used random sampling during the 2018–2019 season abstracted clinical data for 50% of cases among patients aged ≥65 years. Complete clinical data was abstracted for patients in all other age groups.

## eAppendix 2. Primary Outcomes Model Selection

A minimum sufficient set of primary outcome model covariates were derived based on a multi-step process:

1. A literature review was performed of factors associated with social vulnerability and influenza outcomes.
2. Tests were performed (chi-squared for categorical variables, t-tests for continuous variables) for evidence of crude association ($p<0.05$) between each covariate, outcome, and exposure.
3. An assessment for multilinearity of covariates was then performed to identify highly correlated covariates, with additional investigation of any variables with a tolerance level <0.1, variance inflation factor >10, and condition number >15.[1] For any variables identified as collinear, a priori selection was used to decide which variable to include in the model.
4. To eliminate variables from the model that represent statistical noise, forward model selection using Akaike Information Criterion (AIC) values was performed.[2]

Final primary models for ICU admission and IMV/ECMO were adjusted for age group (18–49, 50–64, and ≥65 years), number of categories of medical conditions (0, 1, 2, 3, ≥4 of the following categories: asthma, chronic lung disease, chronic metabolic disease, blood disorders/hemoglobinopathy, cardiovascular disease, neuromuscular disorders, neurologic disorders, history of Guillain-Barré Syndrome, immunocompromising conditions, renal disease, and liver disease), influenza season, female sex, and category of race and ethnicity. Models for death were adjusted for age group, number of categories of medical conditions, type of residence (e.g., private home, group quarters, assisted living), influenza season, female sex, and category of race and ethnicity.

Notably, the inclusion of categories of race and ethnicity as model covariates reflect social constructs that are informed by structural discrimination, and not driven by biological or cultural differences.[3,4] Area-level social vulnerability based on minority status (i.e., all persons except Non-Hispanic White populations) as well as factors associated with structural racism and discrimination (socioeconomic status, housing, education) were also examined in the primary outcome models.[5]


1. *Ender P. Applied Categorical & Nonnormal Data Analysis: Collinearity Issues. [accessed 2023 July 27]; Available from: http://www.philender.com/courses/categorical/notes2/collin.html*
2. *Schonlau M. Model Selection with AIC. 2021 Canadian Stata Conference; 2021; Virtual: StataCorp LLC; 2021.*
3. *Smedley A, Smedley BD. Race as biology is fiction, racism as a social problem is real: Anthropological and historical perspectives on the social construction of race. Am Psychol 2005; 60: 16-26.*
4. *Bailey ZD, Krieger N, Agénor M, Graves J, Linos N, Bassett MT. Structural racism and health inequities in the USA: evidence and interventions. Lancet 2017; 389: 1453-63.*
5. *Adkins-Jackson PB, Chantarat T, Bailey ZD, Ponce NA. Measuring Structural Racism: A Guide for Epidemiologists and Other Health Researchers. Am J Epidemiol 2022; 191: 539-47.*

## eTable 1. FluSurv-NET Counties, 2014-2015 to 2018-2019 Influenza Seasons

| Site | Number of participating counties[a,b] | % state population represented |
|---|---|---|
| Total | 80 | -- |
| California[a] | 3 | 9% |
| Colorado | 5 | 49% |
| Connecticut | 2 | 29% |
| Georgia[a] | 8 | 39% |
| Maryland | 6 | 46% |
| Michigan | 5 | 13% |
| Minnesota[a] | 7 | 55% |
| New Mexico[a] | 7 | 61% |
| New York | 15 | 11% |
| *Albany*[a] | *8* | |
| *Rochester* | *7* | |
| Ohio[a] | 10 | 18% |
| Oregon[a] | 3 | 44% |
| Tennessee | 8 | 26% |
| Utah | 1 | 36% |

[a] For the 2017–2018 season, sites from California, Georgia, New Mexico, Ohio, Oregon, and Albany, New York used a 50% random sample strategy to abstract clinical data from medical records for cases among patients aged 50–64 years and 25% for cases among patients aged ≥65 years. Minnesota conducted random sampling for 50% of cases among patients aged ≥65 years. For the 2018–2019 season, these sites used a 50% random sample strategy to abstract clinical data from medical records for cases among patients aged ≥65 years.
[b] All contributing counties remained the same throughout the five influenza seasons, except for the addition of one Michigan county starting in the 2017–2018 season.

© 2024 Adams K et al. *JAMA Network Open.*

**eTable 2**. Missingness Among Key Analytic Variables

| Variable | Use | No. (col %) missing N=57,964 | Notes |
|---|---|---|---|
| Hospital admission date | Secondary outcomes | 0 (0%) | All FluSurv-NET patients were required to have a date of hospital admission |
| Transferred from another hospital | Descriptive results | 1 (0%) | Recoded as unknown |
| State | Descriptive results | 0 (0%) | |
| County | Descriptive results | 0 (0%) | |
| Age | Descriptive results, primary and secondary outcomes | 0 (0%) | As age ≥18 years was an eligibility criterion, included patients were required to have a documented age. |
| Sex | Descriptive results, primary outcomes | 0 (0%) | |
| Race | Descriptive results, primary outcomes | 1 (0%) | Recoded not specified |
| Ethnicity | Descriptive results, primary outcomes | 2 (0%) | Recoded as not specified |
| Insurance status | Descriptive results | 883/26,729 (3.3%) | Recoded as unknown. Insurance status only collected by FluSurv-NET for the 2017–2018 and 2018–2019 influenza seasons. |
| Residence type | Descriptive results, primary outcomes | 0 (0%) | Missing residential type were excluded under criterion of not having an address type that was residential. |
| Influenza type | Descriptive results | 0 (0%) | |
| Symptom onset date | | 5,482 (9.5%) | Due to the high number of patients missing symptom onset date, this variable was not considered in the selection of secondary outcomes. |
| Respiratory signs/symptoms at admission | Descriptive results | 0 (0%) | ≥1 respiratory sign or symptom was an eligibility criterion, so included patients were required to have these documented. |
| Body mass index (BMI) | Descriptive results | 3,218 (5.6%) | Missingness for BMI due to patients missing weight (n=283) or height (n=2,089) or both (n=846). Coded as unknown for BMI category. |
| Smoker | Descriptive results | 0 (0%) | |
| Alcohol use | Descriptive results | 1 (0%) | Recoded as no/unknown |
| Pre-existing medical conditions | Descriptive results, primary outcomes | 0 (0%) | |
| Intensive care unit admission | Descriptive results, primary outcomes | 0 (0%) | |
| Invasive mechanical ventilation | Descriptive results, primary outcomes | 2 (0%) | Recoded as unknown |

© 2024 Adams K et al. *JAMA Network Open.*

| | | | |
|---|---|---|---|
| ECMO | Descriptive results, primary outcomes | 2 (0%) | Recoded as unknown |
| Death | Descriptive results, primary outcomes | 0 (0%) | All included patients were required to have final outcome (alive/deceased). |
| Received antiviral treatment | Descriptive results, primary outcomes, secondary outcomes | 76 (0.1%) | Recoded as unknown |
| Antiviral start date | Descriptive results, primary outcomes, secondary outcomes | 0/52,585 (0%) | Denominator is among patients receiving influenza antivirals. |
| Received current season influenza vaccine | Descriptive results, primary outcomes, secondary outcomes | 1 (0%) | Recoded as unknown |
| Date of seasonal influenza vaccination | Secondary outcomes | 0/28,643 (0%) | Denominator is among patients receiving a seasonal influenza vaccination. |

© 2024 Adams K et al. *JAMA Network Open.*

## eTable 3. Secondary Study Outcomes

| Outcome | Numerator | Denominator |
|---|---|---|
| ***A. Vaccination*** | | |
| Proportion receiving seasonal influenza vaccine[a] | Number receiving seasonal influenza vaccine during the influenza season[b] that the hospitalization occurred | Number with known influenza vaccination history |
| Proportion vaccinated by the date considered ideal for most people to be vaccinated | Number receiving seasonal influenza vaccine by October 31st of the influenza season that the hospitalization occurred | Number receiving seasonal influenza vaccine during the influenza season that the hospitalization occurred and who have a documented date of vaccination |
| ***B. Treatment*** | | |
| Proportion initiated on recommended in-hospital antivirals[c] | Number receiving recommended antivirals upon or following hospital admission | Number with known influenza treatment history who were not initiated on antivirals prior to hospitalization |
| Proportion with early initiation of recommended in-hospital antivirals | Number receiving recommended in-hospital antivirals within one day following hospital admission | Number receiving recommended in-hospital antivirals |
| Proportion initiated on antivirals prior to hospital admission | Number receiving recommended antivirals prior to hospital admission | Number receiving recommended antivirals |

[a] A FluSurv-NET case is a patient considered to have received the current season influenza vaccine if one or more doses were administered ≥14 days prior to or after an influenza-associated hospitalization.
[b] An influenza vaccine was considered to have been administered during the current influenza season if received starting on July 1st.
[c] Includes neuraminidase inhibitors (NAIs) (Oseltamivir/Tamiflu, Peramivir/Rapivab, and Zanamivir/Relenza) and Baloxavir marboxil (Xofluza) received following hospital admission.

© 2024 Adams K et al. *JAMA Network Open.*

**eTable 4. US Centers for Disease Control and Prevention and Agency for Toxic Substances and Disease Registry Social Vulnerability Index 2018 Variables and Source Census Tables**

| SVI theme | SVI individual variables | Description | Source ACS table |
|---|---|---|---|
| Theme 1: Socioeconomic status | Below poverty | Percentage of persons below poverty estimate | S0601: Selected Characteristics of the Total and Native Populations in the United States |
| | Unemployed | Percentage of civilian (age 16+) unemployed estimate | DP03: Selected Economic Characteristics |
| | Income | Per capita income estimate, 2014–2018 ACS | B19301: Per Capita Income |
| | No high school diploma | Percentage of persons with no high school diploma (age 25+) estimate | S0601: Selected Characteristics of the Total and Native Populations in the United States |
| Theme 2: Household composition & disability | Aged 65 or older | Percentage of persons aged 65 and older estimate | S0101: Age and Sex |
| | Aged 17 or younger | Percentage of persons aged 17 and younger estimate | B09001: Population Under 18 Years by Age |
| | Civilian with a disability | Percentage of civilian Noninstitutionalized population with a disability estimate, 2014–2018 ACS | DP02: Selected Social Characteristics in the United States |
| | Single-parent households | Percentage of single parent households with children under 18 estimate, 2014–2018 ACS | DP02: Selected Social Characteristics in the United States |
| Theme 3: Minority status & language | Minority | Percentage minority (all persons except white, non-Hispanic) estimate, 2014–2018 ACS | B01001H: Sex by Age (White Alone, Not Hispanic or Latino) |
| | Speaks English "Less than well" | Percentage of persons (age 5+) who speak English "less than well" estimate, 2014–2018 ACS | B16005: Nativity by Language Spoken at Home by Ability to Speak English for the Population 5 Years and Over |
| Theme 4: Housing type & transportation | Multi-unit structures | Percentage of housing in structures with 10 or more units estimate | DP04: Selected Housing Characteristics |
| | Mobile homes | Percentage of mobile homes estimate | DP04: Selected Housing Characteristics |
| | Crowding | Percentage of occupied housing units with more people than rooms estimate | DP04: Selected Housing Characteristics |
| | No vehicle | Percentage of households with no vehicle available estimate | DP04: Selected Housing Characteristics |
| | Group quarters | Percentage of persons in group quarters estimate, 2014–2018 ACS | B26001: Group Quarters Population |

Abbreviations: ACS = American Community Survey ; SVI = Social Vulnerability Index

© 2024 Adams K et al. *JAMA Network Open.*

**eTable 5. Mediation Analysis of Significant Associations Found in Primary Analysis, 2014-2015 to 2018-2019 Influenza Seasons, FluSurv-NET**

| | Generalized structural equation modeling[a] | | | | | | | |
|---|---|---|---|---|---|---|---|---|
| | Vaccination status | | Any antiviral receipt | | Number of categories of underlying medical conditions[b] | | Number of respiratory symptoms[c] | |
| | Coefficient (95% CI) | p-value | Coefficient (95% CI) | p-value | Coefficient (95% CI) | p-value | Coefficient (95% CI) | p-value |
| ICU admission | | | | | | | | |
| Overall SVI | | | | | | | | |
| Q2 vs. Q1 | | | | | | | | |
| Direct effect | -0.01 (-0.27–0.24) | 0.93 | -0.01 (-0.27–0.24) | 0.93 | -0.01 (-0.27–0.24) | 0.93 | 0.00 (-0.26–0.25) | 0.97 |
| Indirect effect | 0.00 (0.00–0.00) | 0.51 | 0.00 (0.00–0.00) | 0.78 | 0.00 (0.00–0.01) | 0.17 | 0.00 (0.00–0.00) | 0.74 |
| Total effect | -0.01 (-0.27–0.24) | 0.93 | -0.01 (-0.27–0.24) | 0.93 | -0.01 (-0.26–0.25) | 0.95 | 0.00 (-0.26–0.25) | 0.97 |
| Q3 vs. Q1 | | | | | | | | |
| Direct effect | 0.04 (-0.23–0.30) | 0.80 | 0.04 (-0.23–0.30) | 0.80 | 0.04 (-0.23–0.30) | 0.80 | 0.05 (-0.22–0.32) | 0.74 |
| Indirect effect | 0.00 (0.00–0.00) | 0.88 | 0.00 (0.00–0.00) | 0.84 | 0.01 (0.00–0.01) | 0.17 | 0.00 (0.00–0.00) | 0.78 |
| Total effect | 0.04 (-0.23–0.30) | 0.80 | 0.04 (-0.23–0.30) | 0.80 | 0.04 (-0.23–0.31) | 0.77 | 0.05 (-0.22–0.32) | 0.74 |
| Q4 vs. Q1 | | | | | | | | |
| Direct effect | -0.04 (-0.28–0.21) | 0.77 | -0.04 (-0.28–0.20) | 0.77 | -0.04 (-0.28–0.20) | 0.77 | -0.01 (-0.25–0.24) | 0.96 |
| Indirect effect | 0.00 (0.00–0.00) | 0.93 | 0.00 (0.00–0.00) | 0.65 | 0.01 (0.00–0.02) | 0.15 | 0.00 (-0.01–0.00) | 0.04 |
| Total effect | -0.04 (-0.28–0.21) | 0.77 | -0.04 (-0.28–0.20) | 0.77 | -0.03 (-0.27–0.21) | 0.83 | -0.01 (-0.25–0.24) | 0.94 |
| Theme 1: SES | | | | | | | | |
| Q2 vs. Q1 | | | | | | | | |
| Direct effect | -0.06 (-0.30–0.18) | 0.64 | -0.06 (-0.30–0.18) | 0.64 | -0.06 (-0.30–0.18) | 0.64 | -0.05 (-0.29–0.19) | 0.68 |
| Indirect effect | 0.00 (0.00–0.00) | 0.75 | 0.00 (0.00–0.00) | 0.96 | 0.00 (0.00–0.01) | 0.10 | 0.00 (0.00–0.00) | 0.86 |
| Total effect | -0.06 (-0.30–0.18) | 0.64 | -0.06 (-0.30–0.18) | 0.64 | -0.05 (-0.30–0.19) | 0.66 | -0.05 (-0.29–0.19) | 0.68 |
| Q3 vs. Q1 | | | | | | | | |
| Direct effect | 0.03 (-0.22–0.28) | 0.81 | 0.03 (-0.22–0.28) | 0.81 | 0.03 (-0.22–0.28) | 0.81 | 0.05 (-0.20–0.30) | 0.71 |
| Indirect effect | 0.00 (0.00–0.00) | 0.89 | 0.00 (0.00–0.00) | 0.83 | 0.01 (0.00–0.01) | 0.24 | 0.00 (-0.01–0.00) | 0.17 |
| Total effect | 0.03 (-0.22–0.28) | 0.81 | 0.03 (-0.22–0.28) | 0.81 | 0.036 (-0.22–0.29) | 0.78 | 0.05 (-0.21–0.30) | 0.72 |
| Q4 vs. Q1 | | | | | | | | |
| Direct effect | 0.01 (-0.25–0.28) | 0.93 | 0.01 (-0.25–0.27) | 0.94 | 0.01 (-0.25–0.27) | 0.94 | 0.05 (-0.23–0.32) | 0.74 |
| Indirect effect | 0.00 (0.00–0.00) | 0.56 | 0.00 (0.00–0.00) | 0.67 | 0.01 (0.00–0.02) | 0.08 | 0.00 (0.00–0.00) | 0.09 |
| Total effect | 0.01 (-0.25–0.28) | 0.93 | 0.01 (-0.25–0.27) | 0.94 | 0.02 (-0.24–0.29) | 0.87 | 0.04 (-0.23–0.31) | 0.76 |

© 2024 Adams K et al. *JAMA Network Open.*

| | Generalized structural equation modeling[a] | | | | | | | |
|---|---|---|---|---|---|---|---|---|
| | Vaccination status | | Any antiviral receipt | | Number of categories of underlying medical conditions[b] | | Number of respiratory symptoms[c] | |
| | Coefficient (95% CI) | p-value | Coefficient (95% CI) | p-value | Coefficient (95% CI) | p-value | Coefficient (95% CI) | p-value |
| IMV/ECMO | | | | | | | | |
| Overall SVI | | | | | | | | |
| Q2 vs. Q1 | | | | | | | | |
| Direct effect | -0.03 (-0.13–0.06) | 0.52 | -0.03 (-0.13–0.07) | 0.53 | **-0.03 (-0.13–0.06)** | **0.53** | -0.02 (-0.11–0.08) | 0.76 |
| Indirect effect | 0.00 (0.00–0.00) | 0.28 | 0.00 (0.00–0.00) | 0.76 | **0.01 (0.00–0.01)** | **0.00** | 0.00 (0.00–0.00) | 0.61 |
| Total effect | -0.03 (-0.13–0.06) | 0.52 | -0.03 (-0.13–0.07) | 0.53 | **-0.02 (-0.12–0.07)** | **0.62** | -0.02 (-0.11–0.09) | 0.75 |
| Q3 vs. Q1 | | | | | | | | |
| Direct effect | **0.05 (-0.04–0.15)** | **0.30** | 0.06 (-0.04–0.15) | 0.26 | **0.06 (-0.04–0.15)** | **0.26** | 0.07 (-0.02–0.17) | 0.13 |
| Indirect effect | **0.00 (0.00–0.00)** | **0.02** | 0.00 (0.00–0.00) | 0.84 | **0.01 (0.00–0.01)** | **0.00** | 0.00 (0.00–0.00) | 0.69 |
| Total effect | **0.05 (-0.04–0.15)** | **0.29** | 0.06 (-0.04–0.15) | 0.26 | **0.06 (-0.03–0.16)** | **0.21** | 0.07 (-0.02–0.17) | 0.14 |
| Q4 vs. Q1 | | | | | | | | |
| Direct effect | ***0.15 (0.05–0.24)*** | ***0.00*** | 0.15 (0.06–0.25) | 0.00 | ***0.15 (0.06–0.25)*** | ***0.00*** | ***0.19 (0.09–0.29)*** | ***0.00*** |
| Indirect effect | ***0.00 (0.00–0.00)*** | ***0.00*** | 0.00 (0.00–0.00) | 0.86 | ***0.01 (0.01–0.02)*** | ***0.00*** | ***0.00 (-0.01–0.00)*** | ***0.00*** |
| Total effect | ***0.15 (0.05–0.24)*** | ***0.00*** | 0.15 (0.06–0.25) | 0.00 | ***0.17 (0.07–0.26)*** | ***0.00*** | ***0.19 (0.09–0.29)*** | ***0.00*** |
| Theme 1: SES | | | | | | | | |
| Q2 vs. Q1 | | | | | | | | |
| Direct effect | 0.03 (-0.06–0.12) | 0.55 | 0.03 (-0.06–0.12) | 0.54 | **0.03 (-0.06–0.12)** | **0.54** | 0.04 (-0.05–0.14) | 0.35 |
| Indirect effect | 0.00 (0.00–0.00) | 0.73 | 0.00 (0.00–0.00) | 0.96 | **0.01 (0.00–0.01)** | **0.00** | 0.00 (0.00–0.00) | 0.18 |
| Total effect | 0.03 (-0.06–0.12) | 0.55 | 0.03 (-0.06–0.12) | 0.54 | **0.03 (-0.06–0.13)** | **0.46** | 0.04 (-0.05–0.14) | 0.36 |
| Q3 vs. Q1 | | | | | | | | |
| Direct effect | 0.15 (0.05–0.24) | 0.00 | 0.15 (0.05–0.24) | 0.00 | ***0.15 (0.05–0.24)*** | ***0.00*** | ***0.17 (0.08–0.27)*** | ***0.00*** |
| Indirect effect | 0.00 (0.00–0.00) | 0.80 | 0.00 (0.00–0.00) | 0.80 | ***0.01 (0.00–0.01)*** | ***0.00*** | ***0.00 (-0.01–0.00)*** | ***0.00*** |
| Total effect | 0.15 (0.05–0.24) | 0.00 | 0.15 (0.05–0.24) | 0.00 | ***0.15 (0.06–0.25)*** | ***0.00*** | ***0.17 (0.08–0.26)*** | ***0.00*** |
| Q4 vs. Q1 | | | | | | | | |
| Direct effect | ***0.19 (0.09–0.29)*** | ***0.00*** | 0.20 (0.10–0.30) | 0.00 | ***0.20 (0.10–0.30)*** | ***0.00*** | ***0.24 (0.14–0.34)*** | ***0.00*** |
| Indirect effect | ***0.00 (0.00–0.00)*** | ***0.01*** | 0.00 (0.00–0.00) | 0.47 | ***0.01 (0.01–0.02)*** | ***0.00*** | ***-0.01 (-0.01–0.00)*** | ***0.00*** |
| Total effect | ***0.19 (0.09–0.29)*** | ***0.00*** | 0.20 (0.10–0.30) | 0.00 | ***0.21 (0.11–0.31)*** | ***0.00*** | ***0.24 (0.14–0.34)*** | ***0.00*** |
| Theme 2: Household composition & disability | | | | | | | | |

© 2024 Adams K et al. *JAMA Network Open.*

| | Generalized structural equation modeling[a] | | | | | | | |
|---|---|---|---|---|---|---|---|---|
| | Vaccination status | | Any antiviral receipt | | Number of categories of underlying medical conditions[b] | | Number of respiratory symptoms[c] | |
| | Coefficient (95% CI) | p-value | Coefficient (95% CI) | p-value | Coefficient (95% CI) | p-value | Coefficient (95% CI) | p-value |
| Q2 vs. Q1 | | | | | | | | |
| Direct effect | 0.03 (-0.06–0.12) | 0.53 | 0.03 (-0.07–0.12) | 0.54 | **0.03 (-0.07–0.12)** | **0.55** | 0.04 (-0.06–0.13) | 0.43 |
| Indirect effect | 0.00 (0.00–0.00) | 0.09 | 0.00 (0.00–0.00) | 0.48 | **0.00 (0.00–0.01)** | **0.00** | 0.00 (0.00–0.00) | 0.50 |
| Total effect | 0.03 (-0.06–0.12) | 0.53 | 0.03 (-0.07–0.12) | 0.54 | **0.03 (-0.06–0.13)** | **0.51** | 0.04 (-0.06–0.13) | 0.44 |
| Q3 vs. Q1 | | | | | | | | |
| Direct effect | **0.04 (-0.06–0.14)** | **0.41** | 0.04 (-0.06–0.14) | 0.45 | **0.04 (-0.06–0.13)** | **0.46** | **0.06 (-0.04–0.15)** | **0.27** |
| Indirect effect | **0.00 (0.00–0.00)** | **0.00** | 0.00 (0.00–0.00) | 0.44 | **0.01 (0.00–0.01)** | **0.00** | **0.00 (0.00–0.00)** | **0.02** |
| Total effect | **0.04 (-0.06–0.14)** | **0.41** | 0.04 (-0.06–0.13) | 0.46 | **0.04 (-0.05–0.14)** | **0.39** | **0.05 (-0.04–0.15)** | **0.28** |
| Q4 vs. Q1 | | | | | | | | |
| Direct effect | 0.15 (0.06–0.25) | 0.00 | 0.16 (0.06–0.25) | 0.00 | ***0.16 (0.06–0.25)*** | ***0.00*** | ***0.21 (0.11–0.31)*** | ***0.00*** |
| Indirect effect | 0.00 (0.00–0.00) | 0.11 | 0.00 (0.00–0.00) | 0.48 | ***0.02 (0.01–0.02)*** | ***0.00*** | ***-0.01 (-0.01–0.00)*** | ***0.00*** |
| Total effect | 0.15 (0.06–0.25) | 0.00 | 0.16 (0.06–0.25) | 0.00 | ***0.17 (0.07–0.27)*** | ***0.00*** | ***0.20 (0.10–0.30)*** | ***0.00*** |

***Bold text** = full mediation; ***bold and italicized text*** = partial mediation

Abbreviations: CI = confidence interval; ECMO = extracorporeal membrane oxygenation; ICU = intensive care unit; IMV = invasive mechanical ventilation; SES = socioeconomic status; SVI = Social Vulnerability Index

[a] Models for ICU admission and IMV/ECMO were adjusted for age group (18–49, 50–64, and ≥65 years), number of categories of medical conditions (0, 1, 2, 3, ≥4), influenza season, female sex, and category of race and ethnicity. All models included an interaction term between age group and race and ethnicity. Evidence of mediation was considered when the indirect effects were significant (i.e., 95% confidence intervals did not cross zero), with partial mediation determined when the direct effects were also significant and full mediation when they were non-significant. A review of full analytic output was conducted for any confidence intervals appearing to not cross zero due to rounding.

[b] This model removed underlying medical conditions as a covariate to analyze as a potential mediator.

[c] FluSurv-NET respiratory signs and symptoms include congestion/runny nose, shortness of breath/respiratory distress, cough, sore throat, upper respiratory infections and influenza-like illness (URI/ILI), and/or wheezing occurring within 14 days prior to hospital admission.

© 2024 Adams K et al. *JAMA Network Open.*

**eFigure 1. Random Sampling Scheme for FluSurv-NET 2014-2015 to 2018-2019 Influenza Seasons**

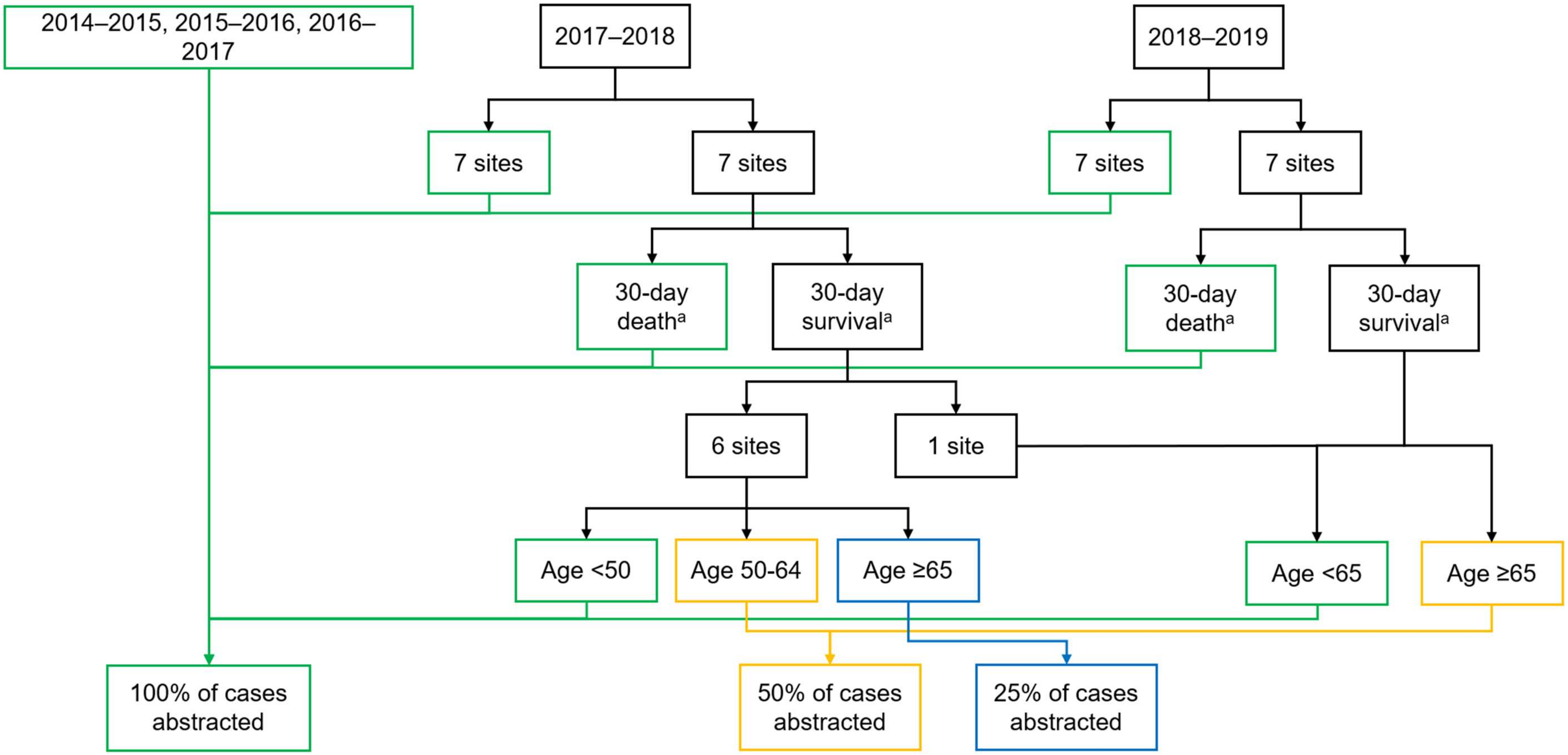


[a] Alive or deceased at 30 days following hospital discharge.

© 2024 Adams K et al. *JAMA Network Open.*

**eFigure 2. Patient Flow Diagram**

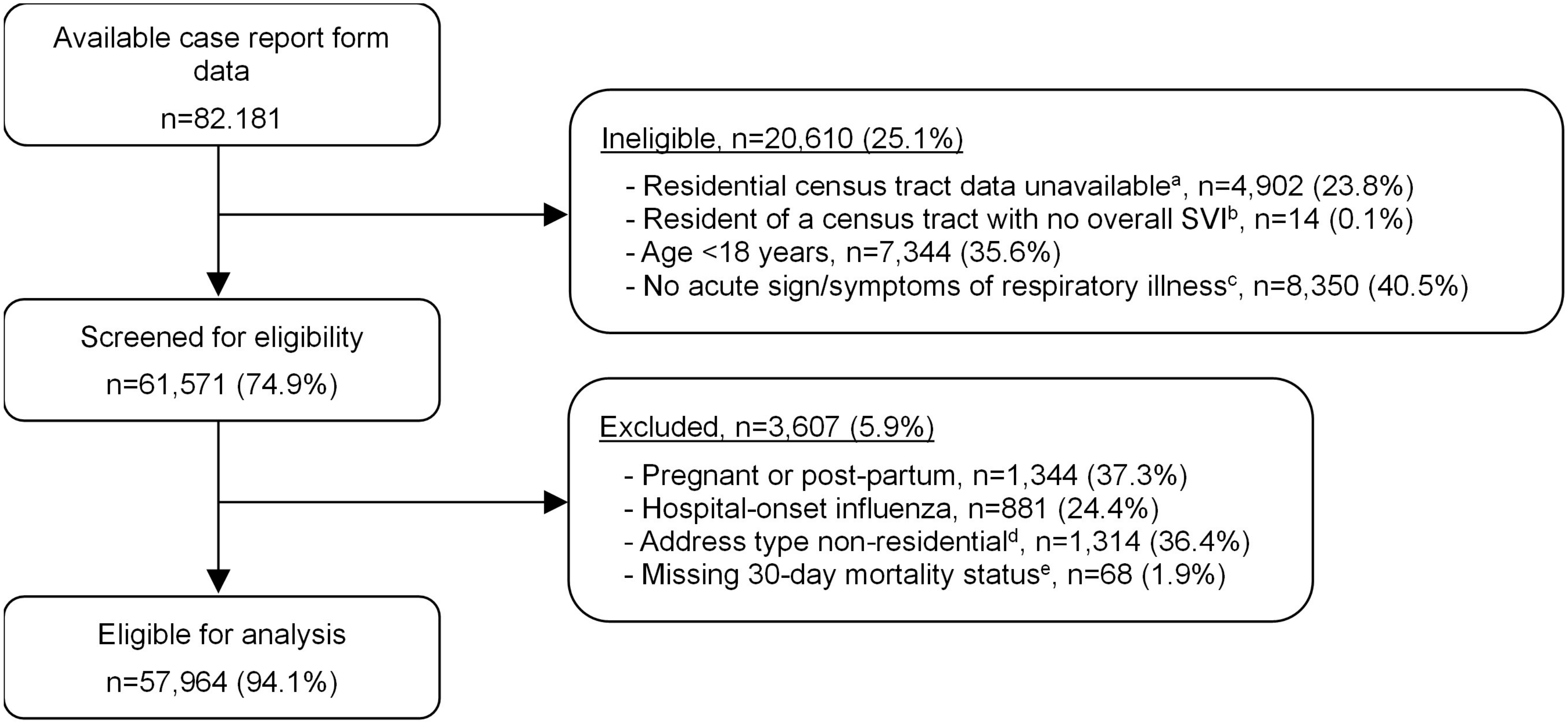


Abbreviations: SVI = Social Vulnerability Index

[a] Contributing FluSurv-NET site was unable to geocode census tract due to lack of patient residential address. Cases among patients using a post office box as an address were not geocoded by contributing sites.

[b] Includes census tracts with zero estimates for total population and tracts that did not provide one or more census indicators used to derive SVI.

[c] FluSurv-NET eligibility criteria includes cases among patients with the non-respiratory influenza symptoms of altered mental status/confusion, fever/chills, and seizures.

[d] Non-residential address types included homeless or homeless shelters, alcohol and drug treatment centers, inpatient rehabilitation facilities, correctional and detention facilities, inpatient psychiatric facilities, and unknown address type.

[e] Cases among patients were excluded if it was unknown whether they were alive or deceased at 30 days following hospital discharge. Cases among patients missing intensive care unit admission status and receipt of invasive mechanical ventilation or extracorporeal membrane oxygenation were only excluded from those respective primary outcome models.

© 2024 Adams K et al. *JAMA Network Open.*

# eFigure 3. Adjusted Prevalence Ratio of Influenza Outcomes Stratified by Age Group, 2014-2015 to 2018-2019 Influenza Seasons, FluSurv-NET

Adjusted PR (95% CI)[a]

Legend: ■ ICU admission; ■ IMV/ECMO; ■ Death; ◆ Q2 versus Q1; ▲ Q3 versus Q1; ● Q4 versus Q1

| SVI theme | Outcome | Quartile | Unweighted No. with outcome/ Unweighted No. in SVI quartile | 18–49 years | Unweighted No. with outcome/ Unweighted No. in SVI quartile | 50–64 years | Unweighted No. with outcome/ Unweighted No. in SVI quartile | ≥65 years |
|---|---|---|---|---|---|---|---|---|
| Overall SVI | ICU admission | 1 | 386/1,998 | Ref. | 620/3,317 | Ref. | 1,514/11,306 | Ref. |
| | | 2 | 413/2,024 | 1.08 (0.95–1.22) | 572/3,250 | 0.95 (0.85–1.05) | 1,259/9,682 | 0.96 (0.89–1.04) |
| | | 3 | 420/2,215 | 1.02 (0.89–1.16) | 633/3,312 | 1.04 (0.93–1.16) | 962/7,267 | 0.97 (0.88–1.06) |
| | | 4 | 516/2,999 | 0.99 (0.87–1.13) | 729/4,282 | 0.97 (0.86–1.09) | 981/6,275 | **1.18 (1.07–1.30)** |
| | IMV/ECMO | 1 | 145/2,000 | Ref. | 271/3,318 | Ref. | 509/11,314 | Ref. |
| | | 2 | 143/2,025 | 1.02 (0.82–1.27) | 232/3,252 | 0.88 (0.74–1.05) | 443/9,689 | 1.02 (0.89–1.17) |
| | | 3 | 160/2,216 | 1.10 (0.89–1.37) | 272/3,316 | 1.02 (0.86–1.21) | 367/7,271 | 1.09 (0.93–1.26) |
| | | 4 | 228/3,001 | **1.34 (1.08–1.67)** | 290/4,284 | 0.87 (0.72–1.06) | 413/6,278 | **1.50 (1.29–1.76)** |
| | Death | 1 | 36/2,000 | Ref. | 118/3,318 | Ref. | 864/11,314 | Ref. |
| | | 2 | 45/2,025 | 1.28 (0.81–2.03) | 108/3,252 | 0.91 (0.70–1.17) | 824/9,689 | 1.06 (0.96–1.18) |
| | | 3 | 37/2,216 | 1.58 (0.73–1.84) | 137/3,316 | 1.22 (0.95–1.56) | 605/7,271 | 1.00 (0.90–1.11) |
| | | 4 | 56/3,001 | 1.29 (0.84–2.00) | 131/4,284 | 0.83 (0.63–1.10) | 500/6,278 | 1.07 (0.94–1.21) |
| Theme 1: SES | ICU admission | 1 | 416/2,220 | Ref. | 663/3,653 | Ref. | 1,784/13,616 | Ref. |
| | | 2 | 444/2,090 | **1.15 (1.01–1.30)** | 621/3,381 | 1.02 (0.93–1.13) | 1,243/9,493 | 1.00 (0.93–1.08) |
| | | 3 | 440/2,227 | 1.08 (0.95–1.22) | 626/3,394 | 1.01 (0.91–1.12) | 932/6,605 | 1.09 (1.00–1.19) |
| | | 4 | 435/2,699 | 0.96 (0.84–1.10) | 644/3,733 | 1.03 (0.91–1.16) | 757/4,816 | **1.21 (1.09–1.34)** |
| | IMV/ECMO | 1 | 162/2,222 | Ref. | 281/3,655 | Ref. | 602/13,626 | Ref. |
| | | 2 | 149/2,091 | 1.01 (0.81–1.26) | 256/3,383 | 1.01 (0.85–1.20) | 431/9,498 | 1.05 (0.92–1.19) |
| | | 3 | 175/2,228 | 1.17 (0.95–1.45) | 247/3,395 | 0.97 (0.81–1.15) | 380/6,611 | **1.32 (1.14–1.51)** |
| | | 4 | 190/2,701 | 1.22 (0.97–1.54) | 281/3,737 | 1.05 (0.87–1.28) | 319/4,817 | **1.56 (1.32–1.84)** |
| | Death | 1 | 44/2,222 | Ref. | 125/3,655 | Ref. | 1,111/13,626 | Ref. |
| | | 2 | 42/2,091 | 1.06 (0.67–1.66) | 129/3,383 | 1.10 (0.86–1.41) | 798/9,498 | 1.03 (0.94–1.13) |
| | | 3 | 43/2,228 | 1.18 (0.75–1.86) | 126/3,395 | 1.09 (0.85–1.39) | 535/6,611 | 1.00 (0.91–1.11) |
| | | 4 | 45/2,701 | 0.99 (0.63–1.55) | 114/3,737 | 0.93 (0.70–1.23) | 349/4,817 | 1.04 (0.91–1.19) |
| Theme 2: Household composition & disability | ICU admission | 1 | 461/2,375 | Ref. | 679/3,764 | Ref. | 1,439/10,318 | Ref. |
| | | 2 | 443/2,321 | 0.99 (0.87–1.11) | 688/3,668 | 1.03 (0.94–1.14) | 1,330/10,276 | 0.95 (0.88–1.03) |
| | | 3 | 434/2,334 | 1.00 (0.88–1.12) | 603/3,338 | 1.00 (0.90–1.11) | 1,114/8,287 | 0.96 (0.88–1.04) |
| | | 4 | 397/2,206 | 0.98 (0.86–1.11) | 584/3,391 | 0.98 (0.88–1.09) | 833/5,649 | 1.08 (0.98–1.19) |
| | IMV/ECMO | 1 | 157/2,377 | Ref. | 285/3,766 | Ref. | 489/10,325 | Ref. |
| | | 2 | 168/2,321 | 1.12 (0.91–1.39) | 285/3,669 | 1.01 (0.86–1.18) | 471/10,281 | 1.03 (0.90–1.18) |
| | | 3 | 170/2,336 | 1.20 (0.97–1.48) | 250/3,339 | 1.00 (0.85–1.19) | 425/8,294 | 1.07 (0.92–1.23) |
| | | 4 | 181/2,208 | **1.38 (1.12–1.70)** | 245/3,396 | 0.98 (0.82–1.17) | 347/5,652 | **1.29 (1.10–1.50)** |
| | Death | 1 | 45/2,377 | Ref. | 136/3,766 | Ref. | 841/10,325 | Ref. |
| | | 2 | 48/2,321 | 1.18 (0.81–1.71) | 134/3,669 | 0.99 (0.79–1.24) | 820/10,281 | 1.00 (0.90–1.10) |
| | | 3 | 39/2,336 | 1.09 (0.70–1.68) | 107/3,339 | 0.89 (0.69–1.15) | 685/8,294 | 1.03 (0.93–1.15) |
| | | 4 | 42/2,208 | 1.12 (0.75–1.68) | 117/3,396 | 0.97 (0.76–1.24) | 447/5,652 | 1.00 (0.89–1.13) |
| Theme 3: Minority status & language | ICU admission | 1 | 211/1,046 | Ref. | 387/2,139 | Ref. | 896/6,901 | Ref. |
| | | 2 | 380/1,941 | 1.03 (0.89–1.19) | 619/3,272 | 1.05 (0.93–1.18) | 1,328/9,941 | 1.02 (0.93–1.11) |
| | | 3 | 569/3,040 | 1.01 (0.88–1.17) | 768/4,176 | 1.08 (0.96–1.21) | 1,366/9,913 | 1.03 (0.94–1.12) |
| | | 4 | 575/3,209 | 1.04 (0.88–1.22) | 780/4,574 | 1.03 (0.91–1.18) | 1,126/7,775 | 1.07 (0.96–1.19) |
| | IMV/ECMO | 1 | 76/1,046 | Ref. | 165/2,139 | Ref. | 311/6,907 | Ref. |
| | | 2 | 152/1,942 | 1.15 (0.89–1.50) | 268/3,276 | 1.07 (0.88–1.31) | 465/9,948 | 1.04 (0.89–1.20) |
| | | 3 | 214/3,043 | 1.15 (0.87–1.51) | 322/4,179 | 1.07 (0.88–1.30) | 494/9,917 | 1.01 (0.86–1.18) |
| | | 4 | 234/3,211 | 1.16 (0.90–1.50) | 310/4,576 | 0.94 (0.75–1.17) | 462/7,780 | **1.22 (1.01–1.46)** |
| | Death | 1 | 20/1,046 | Ref. | 66/2,139 | Ref. | 551/6,907 | Ref. |
| | | 2 | 44/1,942 | 1.32 (0.77–2.25) | 138/3,276 | **1.40 (1.04–1.87)** | 791/9,948 | 0.99 (0.88–1.10) |
| | | 3 | 53/3,043 | 1.21 (0.69–2.12) | 152/4,179 | 1.19 (0.87–1.64) | 820/9,917 | 0.99 (0.89–1.11) |
| | | 4 | 57/3,211 | 1.23 (0.72–2.11) | 138/4,576 | 1.00 (0.71–1.42) | 631/7,780 | 1.02 (0.88–1.17) |
| Theme 4: Housing type & transportation | ICU admission | 1 | 419/2,078 | Ref. | 590/3,188 | Ref. | 1,264/9,209 | Ref. |
| | | 2 | 398/2,203 | 0.92 (0.81–1.04) | 606/3,343 | 1.01 (0.90–1.13) | 1,117/8,461 | 0.97 (0.89–1.05) |
| | | 3 | 433/2,323 | 0.95 (0.84–1.07) | 621/3,516 | 0.98 (0.88–1.09) | 1,150/8,182 | 1.00 (0.92–1.09) |
| | | 4 | 485/2,632 | 0.96 (0.85–1.08) | 737/4,114 | 1.01 (0.90–1.12) | 1,185/8,678 | 1.01 (0.93–1.11) |
| | IMV/ECMO | 1 | 161/2,080 | Ref. | 258/3,194 | Ref. | 430/9,214 | Ref. |
| | | 2 | 158/2,203 | 0.97 (0.79–1.19) | 249/3,344 | 0.98 (0.82–1.16) | 435/8,467 | 1.13 (0.98–1.31) |
| | | 3 | 161/2,324 | 0.94 (0.77–1.15) | 270/3,516 | 0.99 (0.84–1.17) | 394/8,188 | 1.03 (0.88–1.20) |
| | | 4 | 196/2,635 | 1.05 (0.86–1.28) | 288/4,116 | 0.95 (0.80–1.13) | 473/8,683 | **1.17 (1.01–1.36)** |
| | Death | 1 | 40/2,080 | Ref. | 119/3,194 | Ref. | 657/9,214 | Ref. |
| | | 2 | 41/2,203 | 1.00 (0.65–1.54) | 102/3,344 | 0.80 (0.62–1.04) | 674/8,467 | 1.01 (0.90–1.13) |
| | | 3 | 33/2,324 | 0.79 (0.50–1.25) | 124/3,516 | 0.95 (0.73–1.24) | 681/8,188 | 1.02 (0.91–1.14) |
| | | 4 | 60/2,635 | 1.29 (0.87–1.91) | 149/4,116 | 0.89 (0.70–1.15) | 781/8,683 | 1.03 (0.92–1.16) |

Axis (each age group): 0.5 1 1.5 2

## eFigure 4. Adjusted Prevalence Ratio of Influenza Outcomes by SVI Indicator, 2014-2015 to 2018-2019 Influenza Seasons, FluSurv-NET

| SVI theme | Outcome | Indicator | Quartile | Unweighted No. with outcome/ Unweighted No. in SVI quartile | Weighted % (95% CI) | Adjusted PR (95% CI)[a] |
|---|---|---|---|---|---|---|
| Theme 1: SES | ICU admission | 1: Below poverty | 1 | 2,868/18,974 | 14.5 (14.0–15.0) | Ref. |
| | | | 2 | 2,233/14,550 | 14.7 (14.1–15.4) | 1.00 (0.95–1.06) |
| | | | 3 | 1,927/12,217 | 15.1 (14.4–15.7) | 0.99 (0.94–1.05) |
| | | | 4 | 1,977/12,186 | 16.0 (15.3–16.7) | 1.06 (0.99–1.14) |
| | | 2: Unemployment | 1 | 2,743/17,760 | 14.8 (14.3–15.3) | Ref. |
| | | | 2 | 2,339/15,229 | 14.8 (14.2–15.4) | 0.98 (0.93–1.03) |
| | | | 3 | 2,044/12,926 | 15.2 (14.6–15.9) | 1.01 (0.95–1.07) |
| | | | 4 | 1,879/12,012 | 15.2 (14.5–15.9) | 0.99 (0.93–1.06) |
| | | 3: Income | 1 | 2,875/19,544 | 14.0 (13.5–14.5) | Ref. |
| | | | 2 | 2,498/16,545 | 14.6 (14.0–15.1) | 1.01 (0.96–1.07) |
| | | | 3 | 1,875/11,318 | 16.2 (15.5–16.9) | **1.09 (1.02–1.16)** |
| | | | 4 | 1,757/10,520 | 16.3 (15.6–17.1) | **1.10 (1.03–1.19)** |
| | | 4: No HS diploma | 1 | 2,634/17,791 | 14.1 (13.6–14.6) | Ref. |
| | | | 2 | 2,325/15,857 | 14.1 (13.6–14.7) | 0.96 (0.91–1.02) |
| | | | 3 | 2,151/12,811 | 16.3 (15.6–17.0) | **1.10 (1.04–1.17)** |
| | | | 4 | 1,895/11,468 | 16.1 (15.4–16.8) | 1.06 (0.99–1.13) |
| | IMV/ECMO | 1: Below poverty | 1 | 1,045/18,989 | 5.2 (4.9–5.5) | Ref. |
| | | | 2 | 842/14,561 | 5.5 (5.1–5.9) | 1.06 (0.96–1.16) |
| | | | 3 | 758/12,220 | 5.8 (5.4–6.3) | 1.08 (0.98–1.20) |
| | | | 4 | 828/12,194 | 6.6 (6.1–7.1) | **1.21 (1.09–1.35)** |
| | | 2: Unemployment | 1 | 993/17,767 | 5.3 (5–5.6) | Ref. |
| | | | 2 | 870/15,239 | 5.5 (5.1–5.9) | 1.01 (0.92–1.11) |
| | | | 3 | 784/12,932 | 5.7 (5.3–6.1) | 1.05 (0.95–1.16) |
| | | | 4 | 826/12,026 | 6.6 (6.1–7) | **1.21 (1.09–1.35)** |
| | | 3: Income | 1 | 1,042/19,558 | 4.9 (4.6–5.2) | Ref. |
| | | | 2 | 953/16,557 | 5.5 (5.2–5.9) | 1.08 (0.99–1.19) |
| | | | 3 | 734/11,324 | 6.2 (5.7–6.7) | **1.16 (1.05–1.29)** |
| | | | 4 | 744/10,525 | 6.8 (6.3–7.4) | **1.27 (1.13–1.42)** |
| | | 4: No HS diploma | 1 | 963/17,802 | 5.1 (4.8–5.4) | Ref. |
| | | | 2 | 874/15,867 | 5.3 (4.9–5.7) | 1.00 (0.91–1.10) |
| | | | 3 | 866/12,823 | 6.4 (6.0–6.9) | **1.20 (1.09–1.32)** |
| | | | 4 | 770/11,472 | 6.4 (6.0–6.9) | **1.17 (1.04–1.31)** |
| Theme 2: Household composition & disability | IMV/ECMO | 1: ≥65 years | 1 | 1,090/17,277 | 6.0 (5.7–6.4) | Ref. |
| | | | 2 | 980/15,593 | 5.9 (5.6–6.3) | 1.01 (0.92–1.10) |
| | | | 3 | 734/12,806 | 5.5 (5.1–5.9) | 0.96 (0.87–1.07) |
| | | | 4 | 669/12,288 | 5.1 (4.7–5.5) | 0.93 (0.84–1.04) |
| | | 2: <18 years | 1 | 849/15,012 | 5.5 (5.1–5.9) | Ref. |
| | | | 2 | 826/14,359 | 5.5 (5.1–5.9) | 1.00 (0.90–1.10) |
| | | | 3 | 849/13,955 | 5.7 (5.3–6.1) | 1.00 (0.91–1.11) |
| | | | 4 | 949/14,638 | 6.1 (5.7–6.5) | 1.07 (0.97–1.18) |
| | | 3: Disability | 1 | 912/16,070 | 5.3 (4.9–5.6) | Ref. |
| | | | 2 | 956/16,609 | 5.5 (5.1–5.8) | 1.02 (0.93–1.13) |
| | | | 3 | 860/14,223 | 5.8 (5.4–6.2) | 1.09 (0.99–1.20) |
| | | | 4 | 745/11,062 | 6.5 (6.1–7.0) | **1.19 (1.07–1.32)** |
| | | 4: Single-parent HH | 1 | 772/14,268 | 5.1 (4.8–5.5) | Ref. |
| | | | 2 | 810/14,429 | 5.3 (4.9–5.7) | 1.02 (0.93–1.13) |
| | | | 3 | 853/13,543 | 6.1 (5.6–6.5) | **1.14 (1.03–1.26)** |
| | | | 4 | 1,038/15,724 | 6.3 (5.9–6.7) | **1.14 (1.03–1.27)** |

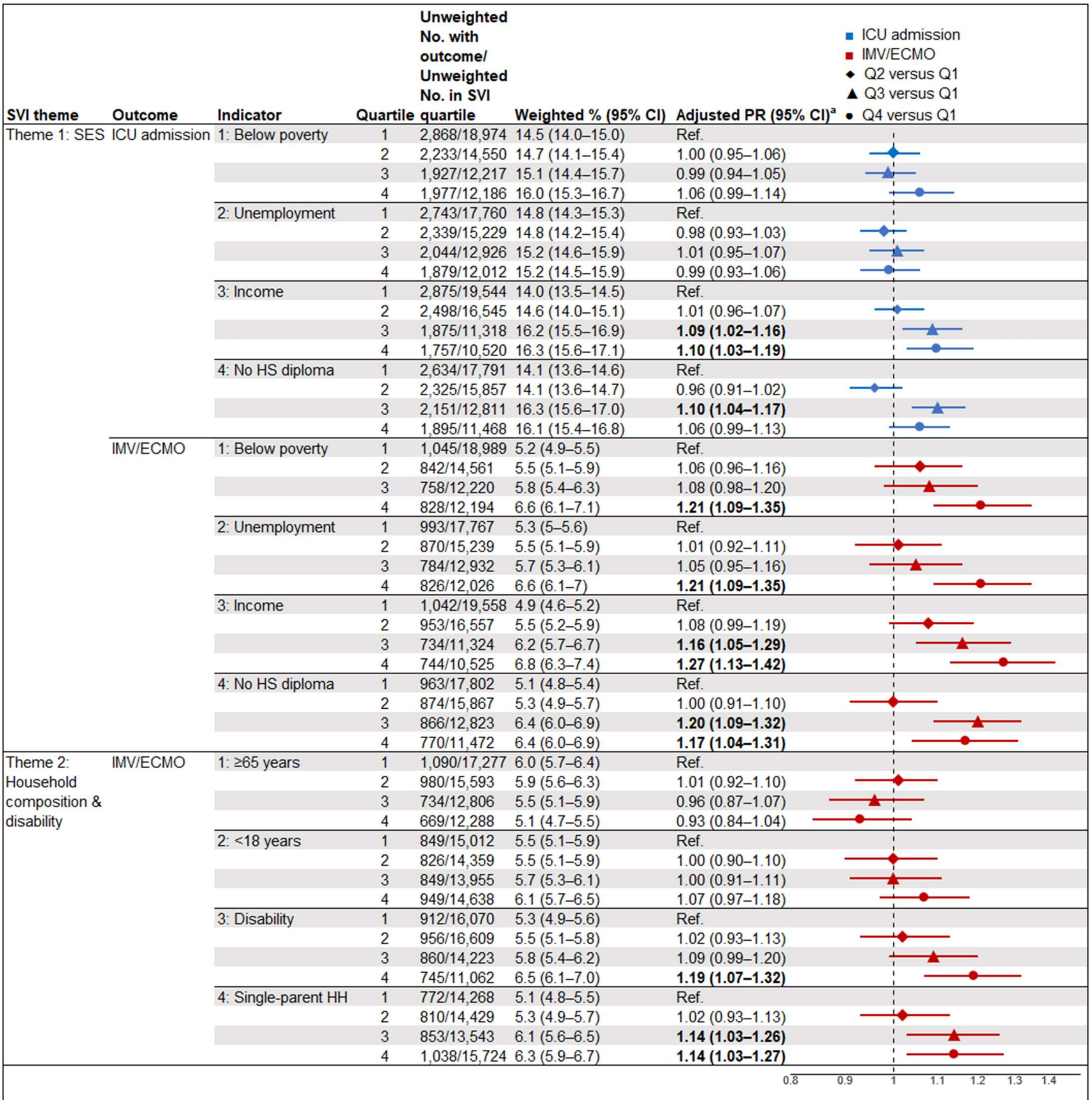


Abbreviations: CI = confidence interval; ECMO = extracorporeal membrane oxygenation; HH = household; HS = high school; ICU = intensive care unit; IMV = invasive mechanical ventilation; PR = prevalence ratio; SES = socioeconomic status; SVI = Social Vulnerability Index

[a] Models for ICU admission and IMV/ECMO were adjusted for age group (18–49, 50–64, and ≥65 years), number of categories of medical conditions (0, 1, 2, 3, ≥4), influenza season, female sex, and category of race and ethnicity. All models included an interaction term between age group and race and ethnicity. Models for ICU admission and IMV/ECMO exclude cases among patients with unknown outcome status.

© 2024 Adams K et al. *JAMA Network Open.*
